# Default egocentrism: an MVPA approach to overlap in own and others’ socio-political attitudes

**DOI:** 10.1093/scan/nsad028

**Published:** 2023-05-27

**Authors:** B Locke Welborn, Macrina C Dieffenbach, Matthew D Lieberman

**Affiliations:** Kairos Research, Dayton, OH 45458, USA; Department of Psychology, University of California, Los Angeles, CA 90095, USA; Department of Psychology, University of California, Los Angeles, CA 90095, USA

**Keywords:** attitudes, projection, theory of mind, MVPA, fMRI

## Abstract

Understanding the socio-political attitudes of other people is a crucial skill, yet the neural mechanisms supporting this capacity remain understudied. This study used multivariate pattern analysis to examine patterns of activity in the default mode network (DMN) while participants assessed their own attitudes and the attitudes of other people. Classification analyses indicated that common patterns in DMN regions encode both own and others’ support across a variety of contemporary socio-political issues. Moreover, cross-classification analyses demonstrated that a common coding of attitudes is implemented at a neural level. This shared informational content was associated with a greater perceived overlap between own attitude positions and those of others (i.e. attitudinal projection), such that higher cross-classification accuracy corresponded with greater attitudinal projection. This study thus identifies a possible neural basis for egocentric biases in the social perception of individual and group attitudes and provides additional evidence for self/other overlap in mentalizing.

## Introduction

There is a long-standing view in social cognition that the way we understand the psychological states and traits of others (i.e. mentalizing) is fundamentally related to the way we understand our own psychological states and traits (self-mentalizing; Ryle, 1949). Some theories prioritize perception and understanding of others, suggesting that awareness of our own mental states emerges when essentially ‘outward-facing’ capacities are turned inward (Bem, 1972) or ‘reflected’ back from others (Shrauger and Schoeneman, 1979; Hardin and Higgins, 1996; Ochsner *et al.*, 2005). Other accounts emphasize that we have privileged access to our own mental states and employ similar mechanisms to understand the minds of others through simulation or projection (Mitchell *et al.*, 2005; Decety and Grèzes, 2006; Goldman, 2006). Yet these accounts share the underlying notion that thinking about the self and thinking about others involve common processes or representations.

The potential overlap in representations of self and others is especially important in the domain of attitude perception. Understanding others’ attitudes in relation to our own is indispensable for establishing coalitions, identifying threats, and resolving conflicts. Yet when we over-rely on the assumption that our attitudes are similar to others, we are subject to distortions: we tend to misunderstand the attitudes of others, systematically overestimating the prevalence of our ‘own’ attitudes within society (i.e. the false consensus effect; Ross *et al.*, 1977; Marks and Miller, 1987) and exaggerating the extremity of opposing views (Robinson *et al.*, 1995). In this ‘naïve realism’ (Griffin and Ross, 1991; Ross and Ward, 1996), we project our own evaluative judgments on to others and on to the world, presuming that any reasonable person would ‘see things’ the way that we do. This attitude misunderstanding can have pernicious consequences, including reactive devaluation of others’ opinions (Maoz *et al.*, 2002; Van Boven *et al.*, 2018) and failures in negotiation (Chamber and De Dreu, 2014).

Suggestively, research in social cognitive neuroscience has identified a general overlap in the neural processes involved in thinking about own and others’ mental states (Lombardo *et al.*, 2010; Van der Meer *et al.*, 2010; Courtney and Meyer, 2020). Quantitative meta-analyses have generally localized this overlap in self- and other-related mental state processing in the default mode network (DMN; see e.g. Schurz *et al.*, 2014 on mentalizing; Northoff *et al.*, 2006 on self-referential processing). Moreover, meta-analyses that explicitly compare self- and other-related processing also find extensive DMN overlap (e.g. Van der Meer *et al.*, 2010; Qin and Northoff, 2011). Could this neural overlap explain, in part, our projective tendencies in attitude (mis)understanding?

Prior evidence has been limited in several important respects that make this question difficult to resolve. First, most work on this topic has employed univariate approaches that examine the presence of common clusters of activation across the two conditions. Better evidence connecting overlapping neural patterns to shared attitudes would be obtained by employing multivariate pattern analysis (MVPA; Weaverdyck *et al.*, 2020), but few studies have explicitly compared self and others with multivariate methods (Oosterwijk *et al.*, 2017; Gilbert and Fung, 2018; Thornton *et al.*, 2019). Second, most mentalizing tasks focus on understanding beliefs rather than attitudes (Denny *et al.*, 2012; Schurz *et al.*, 2014), which may exhibit qualitative differences and are worthy of independent attention.

### Current study

In the present research study, we examine whether thinking about one’s own and others’ socio-political attitudes depends upon common multivoxel pattern information in the DMN/social brain. We further seek to assess whether this neural overlap maps on to presumed similarity of attitudes ascribed to self and others. If the DMN processes own and others’ attitudes using common processes and representations, information relevant to own and others’ attitudes may be successfully decoded from DMN activity using similar classification algorithms and models. We sought to test this hypothesis directly using multivariate pattern analysis (MVPA) of responses in the DMN while participants thought about own and others’ attitudes regarding a variety of contemporary socio-political issues. In our primary analyses, we first sought to determine whether activation patterns in the DMN could predict participants’ own attitudes and their estimates of others’ attitudes. Next, we sought to evaluate own/other overlap through a cross-classification strategy: classification would be trained with data from Own Attitude trials and then tested using data from Other Attitude trials (and vice versa). If these cross-classifications were successful, it would constitute significant evidence that own and others’ attitudes are encoded in the DMN using overlapping processes and/or representations, perhaps forming the basis of egocentric projection of attitudes.

## Methods

### Participants

Twenty-eight participants (21 female and 7 male, age: *M* = 21.89, s.d. = 3.70, range = 18–38) were recruited by email and Internet solicitations from the psychology research subject pool at University of California, Los Angeles (UCLA). All participants had been enrolled as undergraduate students at UCLA for at least two quarters and therefore were likely to possess reasonable levels of exposure to the attitudes of other UCLA students. All participants were compensated $40 for their contribution to this research or received course credit. Participants provided written informed consent approved by the UCLA Institutional Review Board. Five participants’ data were not included in these analyses due to partial acquisition failure or MRI artefacts (final *n* = 23, 18 female and 5 male, age: *M* = 21.14, s.d. = 4.06, range = 18–38).

### Own Attitude and Other Attitude estimation task

Attitude items were derived from a larger set of 155 socio-political issues (e.g. abortion rights and Medicare privatization) that had previously been employed in research on the neural correlates of consensus estimation and the false consensus effect (Welborn *et al.*, 2017; Welborn and Lieberman, 2018). From this larger set, 30 attitude items were selected such that (i) the majority of prior participants were familiar with each item and (ii) the majority of prior participants expressed an attitude for each item (i.e. did not express ‘no opinion’). The complete list of items is included in the Supplementary Table S1.

**Fig. 1.**
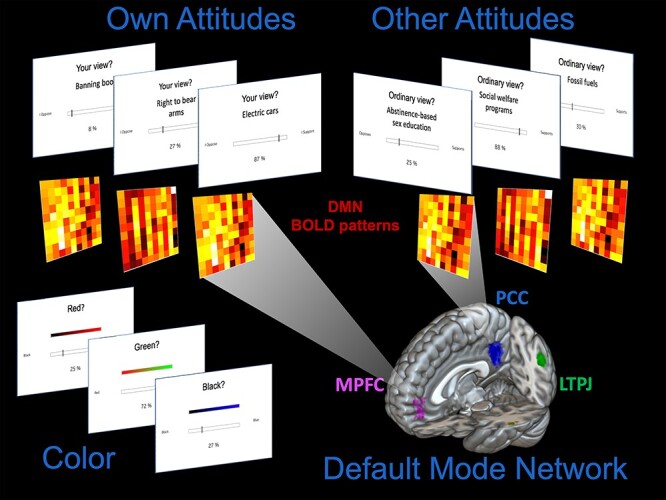
While undergoing fMRI, participants completed an attitude judgment task in which they indicated their own attitudes (Own Attitude trials) and estimated the attitudes of the ordinary person (Other Attitude trials) for a variety of contemporary socio-political issues (see ‘Methods’ section and Supplementary Table S1 for details). Participants responded using a 100-point scale, and their judgments were subsequently divided into evaluative categories reflecting differing degrees of endorsement: Support (≥66), Neutral (>33 and <66) and Oppose (≤33). Participants also performed a color-judgment control task. Univariate BOLD responses and multivariate patterns of BOLD activation were extracted from the DMN for analysis (see ‘Methods’ and ‘Results’ sections). Regions of the DMN were identified based upon automated association test meta-analysis with Neurosynth (www.neurosynth.org) exceeding a threshold of *t* = 7.0. Clusters were identified in the medial prefrontal cortex (MPFC; pink), posterior cingulate/precuneus (PCC; blue), left temporoparietal junction (LTPJ; green), right temporoparietal junction (RTPJ; not shown) and left and right middle temporal gyrus (not shown).

While undergoing functional magnetic resonance imaging (fMRI), participants indicated their Own Attitudes as well as estimates of Others’ Attitudes for each item (Figure 1). To do so, they used an on-screen numeric scale ranging from 0 to 100 in integer increments (anchored at 0—‘Oppose’ and 100—‘Support’). Participants were instructed to use the scale to indicate the intensity of their opinions (and their estimates of others’ opinions) regarding the issues, treating 0 as ‘Complete Opposition’, 25 as ‘Moderate Opposition’, 50 as ‘Neutrality’/‘Neither Supports nor Opposes’, 75 as ‘Moderate Support’ and 100 as ‘Complete Support’. After practicing with a separate set of attitude items (not analysed), participants could use the scale to select any desired response value without difficulty.

On Own Attitude trials, participants were instructed to indicate their own attitude regarding the target issue (with the on-screen query ‘Your view?’). On Other Attitude trials, participants were instructed to indicate the attitude they believe the ordinary UCLA undergraduate student holds regarding the target issue (with the on-screen query ‘Ordinary view?’). In case of ambivalence or uncertainty, participants were instructed to indicate their overall or holistic view (or the overall view of the ordinary UCLA student). Thus, the task (as in prior studies) involves consensus estimation regarding a meaningful in-group (fellow UCLA undergraduate students), although it was not described to participants explicitly in terms of estimating group consensus. Importantly, the task did not involve estimating the attitudes of any particular person but rather those of the generic ‘ordinary UCLA student’. A color-judgment task condition was also included as a non-social control (see Supplementary Materials for details).

To facilitate ease of performance and reduce task demands, trials were grouped by condition into blocks consisting of five trials. Two blocks of each condition were included in each of three functional runs (i.e. 6 blocks and 30 trials per condition per person—yielding 1380 trials for between-subjects MVPA classifications, as outlined below). Block order (i.e. task condition) was randomized within each functional run, subject to the constraint that no condition repeated twice in succession. Each block was preceded by a 2 s cue (e.g. ‘YOUR VIEW’, ‘ORDINARY VIEW’ and ‘COLOR’) to prepare participants for the upcoming block. For each trial, participants had a maximum duration of 10 s to manipulate the scale and confirm their response. Trial presentation was self-paced, with a jittered inter-trial interval commencing immediately after participants’ responses were registered (or 10 s elapsed without a response). Inter-trial jitter was selected from an exponential random distribution with a range of 4–9 s and a mean value of 5 s. The order of items within each condition was randomized across blocks and runs.

Sample size and the number of trials per participant were guided by previous work conducted using a consensus estimation task in the same population (UCLA undergraduates) (Welborn *et al.*, 2017; Welborn and Lieberman, 2018). A larger number of attitude items (trials) per person would have been desirable in order to facilitate within-subjects MVPA classifications, but the number of items was constrained by the variable response time across participants for attitude expression and consensus estimation, as well as the limited duration of available scanning sessions.

### fMRI data acquisition

All imaging data were acquired using a 3.0-Tesla Siemens Prisma scanner at the Ahmanson-Lovelace Brain Mapping Center at UCLA. Across three functional runs, approximately 2100 T2*-weighted echo-planar images were acquired (∼700 per run) during completion of the experimental task, with interleaved acquisitions using a multiband acceleration factor of 8 (slice thickness = 2 mm, gap = 0 mm, 72 slices, TR = 720 ms, TE = 37 ms, flip angle = 52°, matrix = 104 × 104, field of view = 208 mm, phase encoding: anterior-to-posterior). An oblique slice angle was used to minimize signal dropout in ventromedial portions of the brain. In addition, we acquired a T1-weighted magnetically prepared rapid acquisition gradient echo anatomical image (slice thickness = 1 mm, 176 slices, TR = 2530 ms, TE = 3.31 ms, flip angle = 7°, matrix = 256 × 256, field of view = 256 mm).

### fMRI data preprocessing

Structural and functional data were processed using SPM12 (Wellcome Department of Cognitive Neurology, London, UK) (Penny *et al.*, 2007). Within each functional run, image volumes were realigned to correct for head motion, segmented by tissue type, normalized into standard Montreal Neurological Institute (MNI) stereotactic space (resampled at 2 × 2 × 2 mm) and smoothed (6 mm Gaussian kernel, full width at half maximum). Smoothed images were employed for the univariate analyses in order to maximize signal-to-noise ratio; however, unsmoothed images were used for the multivariate analysis (Misaki *et al.*, 2013).

### Region of interest definition for univariate and multivariate analyses

Region of interest (ROI) analyses were conducted to directly assess the overall recruitment and multivariate informational content of the DMN during the task. A priori ROIs were derived from automated meta-analysis through www.neurosynth.org (Yarkoni *et al.*, 2011), using association test masks identified by the term ‘default mode’ and exceeding a *t* threshold of 7.0 (see Figure 1 and Supplementary Materials for details) In the analyses below, the network is considered as a whole (i.e. including the union of all voxels in constituent DMN subregions as features).

For univariate analyses, parameter estimates were extracted from all ROIs using MarsBaR (Brett *et al.*, 2002) for statistical comparisons. Parameter estimates were evaluated at the group level using general mixed-effects models implemented using the lme4 package (Bates *et al.*, 2015) in the R statistical software (http://www.r-project.org/).

### Univariate analytic approach

General linear models (GLMs) were defined for each participant, with trials modelled as variable–duration epochs. Each trial was convolved with the canonical (double gamma) hemodynamic response function. All models controlled for six motion parameters (three translations and three rotations), as well as linear trends and differences between runs. The time series was high-pass filtered using a cutoff period of 128 s, and serial autocorrelations were modelled as an autoregressive AR(1) process. Individual-level statistics were aggregated for group-level comparisons and evaluated with a mixed-effects model. For whole-brain analyses, correction for multiple comparisons was implemented using Gaussian Random Field theory in order to yield a cluster family-wise error of *P* < 0.05 with an initial (voxel-wise) cluster-formation threshold of *P* < 0.001.

### Multivariate analytic approach

In order to conduct multivariate pattern analysis of brain imaging data from the task, a series of beta images were first derived from univariate GLMs (Rissman *et al.*, 2004). Separate models were constructed for ‘Own Attitude’ and ‘Other Attitude’ trials for each run to avoid possible collinearity between responses to the same or similar attitudes across targets. Thus, for example, to derive a beta series for the ‘Own Attitude’ condition, individual parameters were modelled for each trial from the ‘Own Attitude’ condition, while all trials in the ‘Other Attitude’ condition were aggregated under a single parameter. Models also included six motion-related parameters, as for univariate GLMs (see above). Data from the beta series for each subject were imported into Python 3.7.6 using nibabel version 2.1.0 (https://github.com/nipy/nibabel/releases/tag/2.1.0) and extracted for DMN voxels. Finally, data were concatenated across subjects for between-subjects multivariate analyses. Within-subject classifications would suffer from the small number of attitude trials available for each participant (30 each for ‘Own Attitude’ and ‘Other Attitude’ conditions); between-subject analyses can exploit all 1380 trials in the sample (suitably divided into training and testing sets as noted below).

Between-subject multivariate classification analyses were conducted using scikit-learn version 0.20.2 (Pedregosa *et al.*, 2011) pipelines employing three steps: (I) missing voxel imputation, (II) standard scaling and (III) support vector machines (SVM) classification with a radial basis function (RBF) kernel. Missing voxel imputation replaced missing values with the mean value from each voxel. Standard scaling mean centred data from each voxel and divided raw values by their standard deviation. Finally, SVM classification assigned a class label to each case (i.e. trial) within the dataset, using an RBF with default cost and gamma hyperparameters (*C* = 1, gamma = 1/*n*_features). Importantly, each of the above steps was performed within cross-validation folds, so that there would be no data leakage from the training to the testing dataset.

Cross-validation accuracy was assessed using a group shuffle split strategy. Groups of three participants at a time were iteratively held out as test sets, with the remaining 20 participants employed as a training set, over a total of 100 iterations per analysis (see Supplementary Methods for details). Permutation testing was employed to determine whether cross-validation classifier accuracy differed significantly from chance, with 1000 permutations per analysis. For each permutation, dataset class labels were randomly permuted within participants, and the classification algorithm was repeated. The *P*-value was then estimated from the proportion of permutations for which classifier accuracy exceeded the accuracy with non-permuted (true) labels (Ojala and Garriga, 2010). In all cases, balanced accuracy scores were used (in which accuracy for a class is inversely weighted by class frequency) so that a classification cannot emerge as significant simply by virtue of predicting the most frequent class (‘Support’, for the present analyses).

Searchlight MVPA analyses were performed within the DMN in order to spatially localize voxels associated with Own and Other Attitude information. A 6 mm sphere was iterated through the DMN, and only patterns from grey matter voxels were analysed. Parameters for the searchlight MVPA were identical to those employed for the ROI as a whole, except that a leave-one-subject-out cross-validation strategy was used to complete the analysis within a practicable duration. Balanced accuracy scores were computed for each voxel, and permutation testing was used again to assess significance.

## Results

### Behavioural overlap in responses to Own and Other Attitude items

**Fig. 2.**
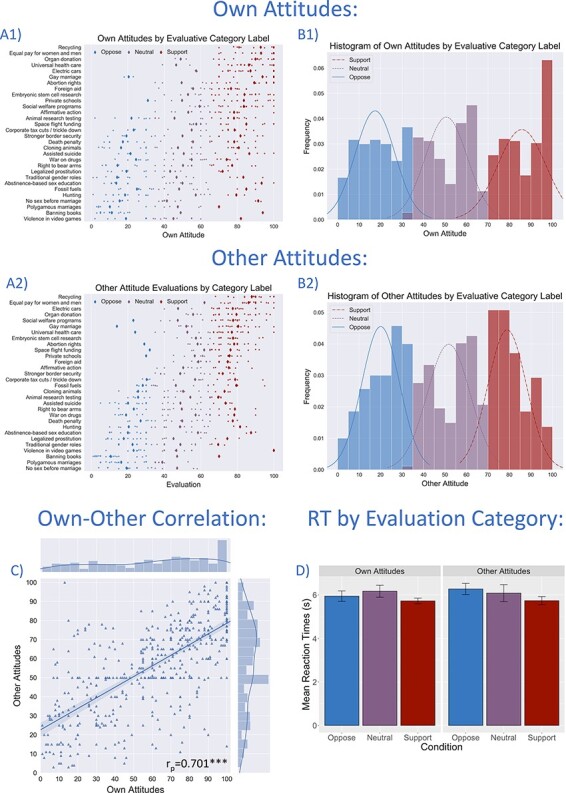
Own Attitudes and estimates of Other Attitudes varied across both participants and attitude items (issues). Responses for Own and Other Attitudes were divided into Oppose (≤33), Neutral (>33 and <66) and Support (≥66) evaluative categories. The heterogeneity of attitudinal responses is depicted in panels A1 (Own Attitudes) and A2 (Other Attitudes) in the form of strip plots. Large diamonds indicate the sample means for each attitude item, split by category. Panels B1 and B2 display (for Own and Other Attitudes, respectively) histograms of attitude scores by evaluative categories, along with estimated underlying Gaussian distributions. The over-representation of Own Attitudes that were fully endorsed (scale value 100) is evident. Panel C depicts the strong correlation between Own Attitude and Other Attitude values across the sample, suggestive of overlap in underlying psychological representations. Panel D plots mean reaction times for Own and Other Attitudes for each evaluative category with 95% confidence intervals. Support judgments were slightly faster than other evaluative categories, but overall reaction times are comparable (see also Supplementary Materials and Supplementary Table S1).

Over the course of the experiment, for each of 30 socio-political attitudes, participants indicated (i) their own attitudes and (ii) their estimates of the attitude of an ordinary UCLA undergraduate (Figure 2). These ratings were made using a 100-point on-screen scale ranging from Complete Opposition (scale value ‘0’) through Neutrality (scale value ‘50’) to Complete Support (scale value ‘100’). These responses, hereafter referred to as Own Attitudes and Others’ Attitudes, form the basis of the following analyses, which examine overlap at the attitudinal level and in the underlying neural correlates.

Across all items and all subjects, a strong association was observed between Own Attitude values and corresponding Other Attitudes (*r* = 0.701, *P* < 0.001, Figure 2C; mixed-effects model with participants as random factor: β = 0.559, *P* < 0.001). Participants’ consensus estimates overestimated support for their own attitudes (relative to the actual sample mean attitude), on average, by 11.36 points out of the 100 point scale (95% CI [9.32, 13.40]), in line with our prior work (cf., mean overestimate of 12.17 in Welborn *et al.*, 2017). These results suggest that projection from self to others is robust for our sample, consistent with previous literature on egocentrism and consensus estimation bias (Krueger and Clement, 1994).

To assess potential overlap in psychological and neural processing of Own and Others’ Attitudes using multivariate classification techniques, items were divided into three groups, reflecting evaluative categories of Opposition (Own/Other score ≤33), Neutrality (33 < Own/Other score < 66) and Support (Own/Other score ≥66). Figure 2 displays histograms of the frequencies of attitude items in each category, plotted separately for Own Attitudes (B1) and Other Attitudes (B2). Attitude items are approximately equally distributed between the three categories, with the pronounced exception of a surplus of Own Attitude items indicating complete support. Classification analyses reported later are thus performed with and without these strongly supportive attitudes, which might plausibly constitute a separate category phenomenologically. Figure 2 also displays the heterogeneity of endorsement across different attitude items in the form of strip plots for each issue (A1 and A2). Reaction times did not differ dramatically between conditions (means were all between 5.70 and 6.30 s). For details, see Supplementary Materials and Supplementary Table S1.

### Univariate analysis of DMN activity to own and others’ attitudes

ROI analyses were conducted on the DMN in order to assess (i) whether the DMN would be more responsive to attitude judgments (Own/Other items) than to control judgments (color items), (ii) whether DMN activation would differ between Own and Other Attitude judgment and (iii) whether DMN activation would differ between evaluative categories (Oppose, Neutral and Support). The results (see Supplementary Results) confirm that the DMN was similarly responsive to Own and Other Attitude judgment but suggest that the mean level of hemodynamic activity in the DMN does not differ based upon evaluative category. Whole-brain analyses provided largely consistent evidence, with similar responses in DMN regions to Own and Other Attitudes (see Supplementary Results and Supplementary Tables S2 and S3 and Figures S2 and S3). Overall, the processes engaged by the DMN when thinking about supportive, neutral and opposed attitudes may not differ dramatically for self and others. However, the underlying patterns of hemodynamic activity, potentially reflecting representations of social attitudes, may nevertheless differ between attitudes even in the absence of differences in the mean hemodynamic response.

### MVPA of Own Attitudes

**Fig. 3.**
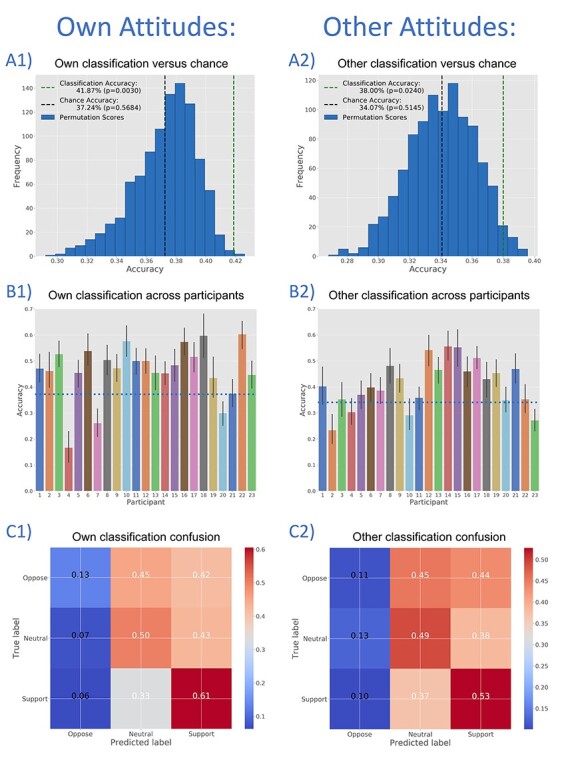
Three-way classifications for Own Attitudes (panels A1, B1 and C1) and Other Attitudes (panels A2, B2 and C2) indicate that BOLD activation partners in the DMN contain information relevant to evaluative categories (Oppose, Neutral and Support). Panels A1 and A2 show cross-validated classification accuracy *vs* a histogram of permutation test accuracy scores. Panels B1 and B2 show classification accuracy across study participants, with above-chance accuracy for most individuals. Panels C1 and C2 show normalized confusion matrices for Own and Other Attitude classifications, with better performance for Support and Neutral items than for Oppose items.

MVPAs in the DMN were conducted to assess whether Own and Others’ attitudes would exhibit similar properties. First, we sought to determine whether DMN activity contained information relevant to Own Attitude positions. Thus, a three-way classification was performed, attempting to identify attitudes in Support, Oppose, and Neutral categories. Classification was successful for Own Attitudes, with a mean accuracy of 41.87% (*P* = 0.003; chance accuracy: 37.24%; Figure 3A1). Classification accuracy is above chance for the majority of participants (Figure 3B1) and attitude items (Supplementary Figure S4A). In the absence of 100-point (full support) items, classification accuracy was slightly improved at 42.42%. These results suggest that DMN activity encodes the basic features of the evaluative content of one’s own attitudes and that this encoding is at least somewhat consistent across attitudes and persons (enabling between-subject classification).

Examination of classifier confusion between evaluative categories (i.e. Support, Neutral, and Oppose; Figure 3C1) shows that Support items and Neutral items are identified relatively well (∼61% and 50% true positive rate, or ‘recall’, respectively) compared to Oppose items (14%). That is, of the items actually supported by participants, 61% were correctly assigned to the Support category by the classifier; of the items that were actually neutral, 50% were correctly assigned to the Neutral category; of the items actually opposed, only 14% were correctly assigned to the Oppose category. Overall, classification was not ‘confused’ by the Support and Neutral items, mostly assigning them to the correct category; in contrast, Oppose items were often assigned inappropriately to the Neutral or Support categories.

**Fig. 4.**
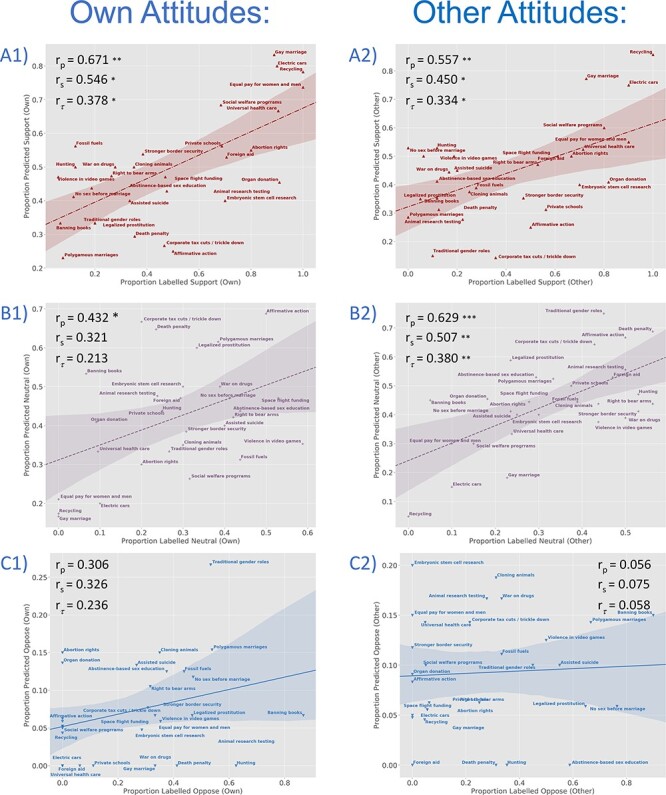
To further assess and better visualize the classification analyses depicted in Figure 3, we evaluate the strength of association, across attitude items, between the proportion of judgments predicted by the classifier to be in a given evaluative category (i.e. Support, Neutral, and Oppose) and the proportion that actually are in that category. Because the small number of attitude items (30) increases the leverage of extreme data points and the influence of possible outliers, we report Spearman’s (*r*_s_) and Kendall’s Tau-b (*r*_τ_) coefficients in addition to Pearson’s coefficients (*r*_p_). Associations are plotted separately for each target (Own, left panels; Other, right panels) and for each category [Support (A1 and A2), Neutral (B1 and B2) and Oppose (C1 and C2)]. This avoids over-plotting (though note the differing *y*-axis scales) and allows for separate evaluation of each category. Please also note that these analyses are not independent of the classifications depicted in Figure 3 and are intended to further visualize these classifications rather than as completely separate tests. With this caveat, it is interesting to note that these associations are generally significant for Support and Neutral attitudes but not for Oppose. *P < 0.05, **P < 0.01, ***P < 0.001. (A1) Own Support: *r*_p_ = 0.671, *P* < 0.001; *r*_s_ = 0.546, *P* = 0.002; *r*_τ_ = 0.378, *P* = 0.008. (A2) Other Support: *r*_p_ = 0.557, *P* = 0.001; *r*_s_ = 0.450, *P* = 0.012; *r*_τ_ = 0.334, *P* = 0.010. (B1) Own Neutral: *r*_p_ = 0.432, *P* = 0.017; *r*_s_ = 0.321, *P* = 0.084; *r*_τ_ = 0.213, *P* = 0.104. (B2) Other Neutral: *r*_p_ = 0.629, *P* < 0.001; *r*_s_ = 0.507, *P* = 0.004; *r*_τ_ = 0.380, *P* = 0.004. (C1) Own Oppose: *r*_p_ = 0.306, *P* = 0.100; *r*_s_ = 0.326, *P* = 0.079; *r*_τ_ = 0.236, *P* = 0.088. (C2) Other Oppose: *r*_p_ = 0.056, *P* = 0.769; *r*_s_ = 0.075, *P* = 0.691; *r*_τ_ = 0.058, *P* = 0.664.

To further illustrate the relationship between evaluative categories and classification decisions, Figure 4 plots, for each attitude item (e.g. ‘Assisted Suicide’ and ‘Abortion rights’), the proportion of participants who endorsed a given position (e.g. ‘Support’) against the proportion predicted to endorse that position by the classifier (note the differing scales for each position). These plots show that for most categories, as an attitude item is psychologically more likely to fall into a given evaluative category (for our sample), the classifier is more likely to assign that category appropriately. Thus, universal health care is highly likely to be labelled ‘support’ by participants and highly likely to be labelled ‘support’ by the classifier. Note that this result is not independent of the permutation test reported earlier and depicted in Figure 3A and reflects further visualization/assessment of these results rather than a completely separate analysis. With this caveat, correlations between the proportion of participants endorsing a given position and the proportion predicted to do so are significant for Support and Neutral items, though not for Oppose items (Figure 4).

### MVPA of others’ attitudes

Next, we sought to determine whether DMN activity also contains information relevant to Other Attitude judgments. Again, a three-way classification was performed, with the goal of identifying attitudes in Support, Oppose, and Neutral categories. For Other Attitudes, this reflected the participant’s judgment that the ordinary person would support, oppose, or feel neutral about a given attitude item. Classification was also successful for Other Attitudes, with a mean accuracy of 38.00% (*P* = 0.024, chance accuracy: 34.07%, Figure 3A2; mean accuracy 37.81% without 100-point responses). Classification accuracy is above chance for the majority of participants (Figure 3B2) and attitude items (Supplementary Figure S4B). These results suggest that DMN activity also encodes the evaluative content of Others’ attitudes and that this encoding may similarly generalize over persons to some extent.

Examination of classifier confusion (Figure 3C2) reveals that, as for Own Attitude classification, Support and Neutral items are identified relatively well (true positive rate of approximately 53% and 49%, respectively), while Oppose items are identified less effectively (11%). Figure 4 also shows that, generally speaking, as participants were more likely to think that the ordinary person would endorse a given position, the classifier was more likely to assign the Other Attitude to that category appropriately [Support (A2), Neutral (B2) and Oppose (C2)]. Correlations are significant for Support and Neutral items but not for Oppose items (Figure 4).

### Cross-classification MVPA of own and others’ attitudes

**Fig. 5.**
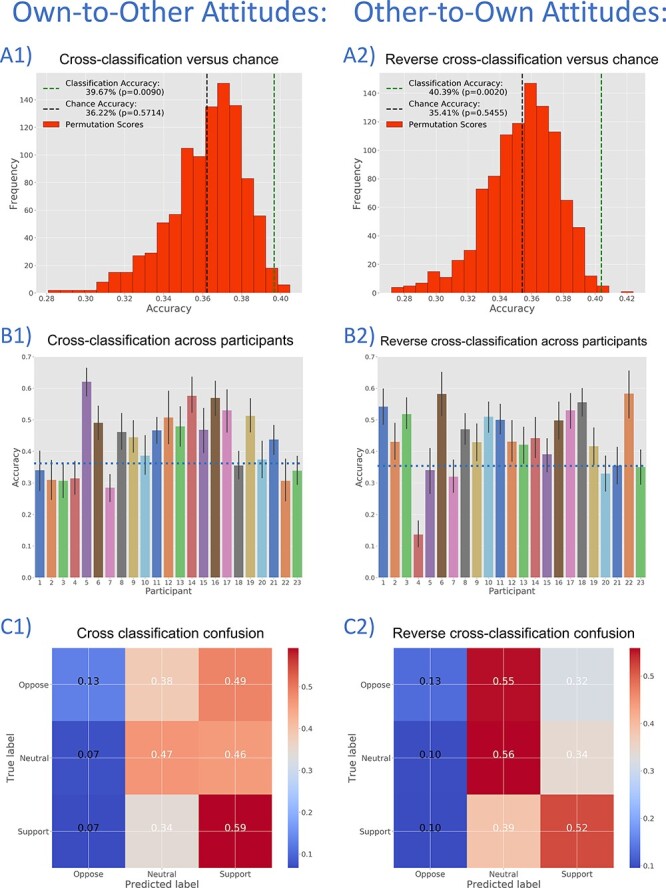
Three-way classifications for Own-to-Other (panels A1, B1 and C1) and Other-to-Own (panels A2, B2 and C2) cross-classification analyses. These analyses train on Own Attitude data and test on Other Attitude data (and vice versa). Panels A1 and A2 show cross-validated classification accuracy *vs* a histogram of permutation test accuracy scores. Panels B1 and B2 show classification accuracy across study participants, with above-chance accuracy for most individuals. Panels C1 and C2 show normalized confusion matrices for Own-to-Other and Other-to-Own classifications.

**Fig. 6.**
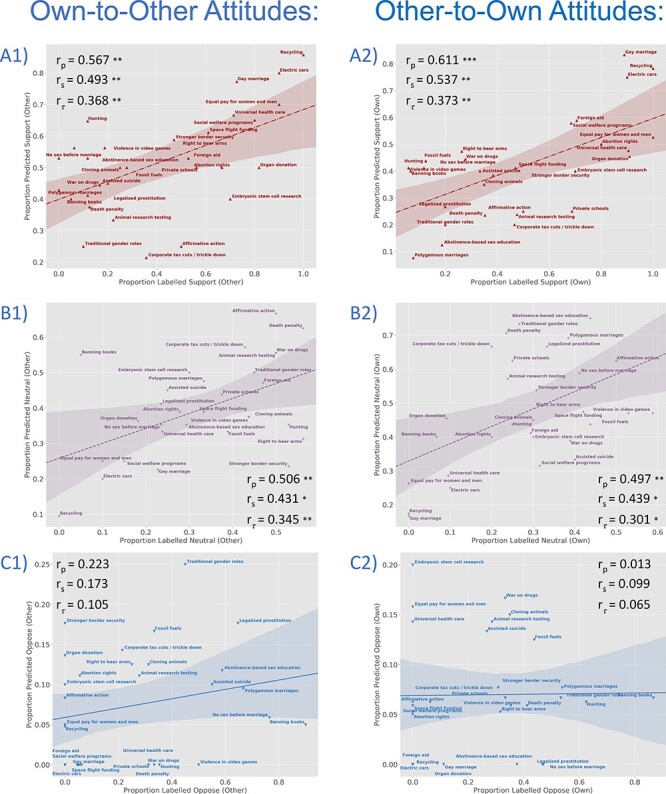
To further assess and better visualize the cross-classification analyses depicted in Figure 5, we evaluate the strength of association, across attitude items, between the proportion of judgments predicted by the classifier to be in a given evaluative category (i.e. Support, Neutral, and Oppose) and the proportion that actually are in that category. Because the small number of attitude items (30) increases the leverage of extreme data points and the influence of possible outliers, we again report Spearman’s (*r*_s_) and Kendall’s Tau-b (*r*_τ_) coefficients in addition to Pearson’s coefficients (*r*_p_). Associations are plotted separately for each target (Own-to-Other, left panels; Other-to-Own, right panels) and for each category [Support (A1 and A2), Neutral (B1 and B2) and Oppose (C1 and C2)]. Please note that these analyses are not independent of the classifications depicted in Figure 5 and are intended to further visualize these classifications rather than as completely separate tests. With this caveat, it is interesting to note (as in Figure 4) that these associations are generally significant for Support and Neutral attitudes but not for Oppose. *P < 0.05, **P < 0.01, ***P < 0.001. (A1) Own-to-Other Support: *r*_p_ = 0.567, *P* = 0.001; *r*_s_ = 0.493, *P* = 0.006; *r*_τ_ = 0.368, *P* = 0.005. (A2) Other-to-Own Support: *r*_p_ = 0.611, *P* < 0.001; *r*_s_ = 0.537, *P* = 0.002; *r*_τ_ = 0.372, *P* = 0.004. (B1) Own-to-Other Neutral: *r*_p_ = 0.506, *P* = 0.04; *r*_s_ = 0.431, *P* = 0.017; *r*_τ_ = 0.345, *P* = 008. (B2) Other-to-Own Neutral: *r*_p_ = 0.497, *P* = 0.005; *r*_s_ = 0.439, *P* = 0.015; *r*_τ_ = 0.301, *P* = 0.021. (C1) Own-to-Other Oppose: *r*_p_ = 0.223, *P* = 0.237; *r*_s_ = 0.173, *P* = 0.361; *r*_τ_ = 0.105; *P* = 0.447. (C2) Other-to-Own Oppose: *r*_p_ = 0.013, *P* = 0.945; *r*_s_ = 0.099, *P* = 0.640; *r*_τ_ = 0.065, *P* = 0.642.

In order to directly test whether or not the information content used in Own and Other classifications is common or distinct, we performed cross-classification analyses. First, we trained an SVM classifier on data and category labels associated with Own Attitudes, and then tested this classifier on data and category labels associated with Other Attitudes. This Own-to-Other cross-classification achieved an accuracy of 39.66% (*P* = 0.009; chance accuracy: 36.22%, Figure 5A1; mean accuracy was 39.26% without 100-point responses). Own-to-Other cross-classification accuracy is above chance for the majority of participants (Figure 5B1) and attitude items (Supplementary Figure S4C). The proportion of attitudes assigned by the classifier to each category also tracks with the number of Other Attitudes actually so labelled by participants (see Figure 6 A1, B1 and C1).

Next, we trained and tested a classifier in the other ‘direction’, training on data and category labels associated with Other Attitudes and testing on data and labels associated with Own Attitudes. This reverse cross-classification achieved a comparable accuracy of 40.38% (*P* = 0.002; chance accuracy: 35.41%; Figure 5A2; mean accuracy 40.11% without 100-point items). The Other-to-Own cross-classification accuracy is above chance for the majority of participants (Figure 5B2) and attitude items (Supplementary Figure S4D). The proportion of attitudes assigned to each category also tracks with the number of Own Attitudes so labelled by participants (Figure 6A2, B2 and C2).

The results of Own-to-Other and Other-to-Own cross-classifications suggest that the information employed by the classifiers in assigning items to evaluative categories is comparable for Own and for Other judgments. While there are certainly instances in which own and others’ attitudes are likely to be processed very differently, in the present study, DMN activity seems to reflect common encoding and/or processing with respect to the evaluative categories examined.

**Fig. 7.**
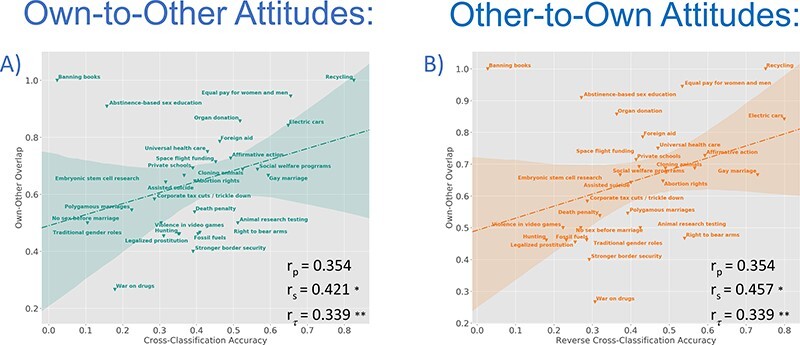
Associations between accuracy and proportion of Own–Other Attitude overlap across attitude items were assessed for Own-to-Other (A) and Other-to-Own (B) cross-classifications. Strength of associations is indicated for Pearson (*r*_p_), Spearman (*r*_s_) and Kendall’s Tau-b (*r*_τ_) coefficients. Accuracy scores for each attitude item are inversely weighted by maximal class frequency (comparable to the balanced weighting scheme used to assess general classifier accuracy) to avoid possible bias in favour of attitudes that are more homogenous across participants (e.g. most participants indicating ‘Support’). *P < 0.05, **P < 0.01. (A) Own-to-Other cross-classification accuracy is positively associated with own–other overlap when assessed by Spearman’s and Kendall’s Tau coefficients (*r*_s_ = 0.421, *P* = 0.020; *r*_τ_ = 0.339, *P* = 0.009) but only marginal with Pearson’s coefficient (*r*_p_ = 0.354, *P* = 0.055). Associations unweighted for class frequency are significant for all three measures (*r*_p_ = 0.476, *P* = 0.008; *r*_s_ = 0.407, *P* = 0.026; *r*_τ_ = 0.330, *P* = 0.011). (B) Other-to-Own ‘reverse’ cross-classification accuracy is also positively associated with own–other overlap when assessed by Spearman’s and Kendall’s Tau coefficients (*r*_s_ = 0.457, *P* = 0.011; *r*_τ_ = 0.339, *P* = 0.009) but marginal with Pearson’s (*r*_p_ = 0.354, *P* = 0.055). Associations unweighted for class frequency are significant for all three measures (*r*_p_ = 0.494, *P* = 0.006; *r*_s_ = 0.517, *P* = 0.003; *r*_τ_ = 0.409, *P* = 0.0002).

Does common processing for own and others’ attitudes at the neural level help explain the overlap at the behavioural level (i.e. projection)? To address this question, we sought to assess the association between cross-classification accuracy and own–other attitudinal overlap. Common processes or representations should best facilitate accurate cross-classification for attitude items where own and others’ attitudes are perceived to be similar but may not help (or may even hinder) cross-classification when own and others’ attitudes are perceived to diverge. As depicted in Figure 7, both Own-to-Other and Other-to-Own cross-classification accuracies were significantly associated with attitude overlap (Own-to-Other: *r*_s_ = 0.421, *P* = 0.020; *r*_τ_ = 0.339, *P* = 0.009, *r*_p_ = 0.354, *P* = 0.055; Other-to-Own: *r*_s_ = 0.457, *P* = 0.011; *r*_τ_ = 0.339, *P* = 0.009; *r*_p_ = 0.354, *P* = 0.055, see Supplementary Methods).^1^ These results suggest that common processing of Own and Other Attitudes may contribute to the observed prevalence of egocentric projection in the perception of group attitudinal positions.

### Searchlight MVPA of own and others’ attitudes within the DMN

**Fig. 8.**
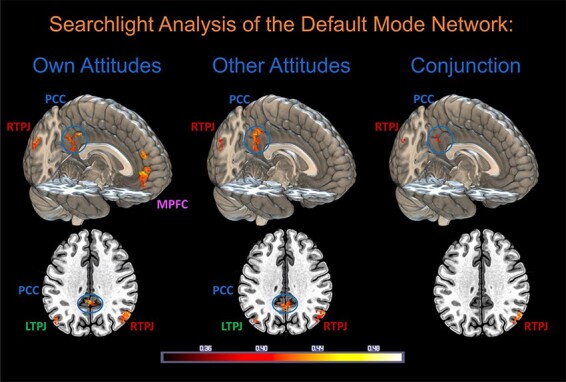
Searchlight MVPA analysis of the DMN reveals regions in which localized patterns of BOLD activity could successfully classify trials between evaluative categories (Support, Neutral, and Oppose) for Own Attitudes, Other Attitudes, and both (conjunction). Significant clusters were identified in all regions of the DMN. Searchlight analysis used the same radial basis function SVM algorithm as employed for the whole DMN, iterating over 6 mm spheres centred around DMN voxels. Only signals from grey matter voxels were analysed. Permutation testing (with 1000 permutations) was used to derive an empirical *P*-value for classification accuracy at each voxel; only voxels significant at *P* < 0.001 (uncorrected), with a cluster size *k* ≥ 15, are displayed. Axial slices are at MNI *z* = 36.

To spatially localize subregions within the DMN that may be especially critical to the processing of own and others’ attitudes, searchlight MVPA was conducted within this volume. The results indicate that information relevant to own and others’ attitudinal positions is encoded in multiple DMN subregions, including the medial prefrontal cortex (MPFC), posterior cingulate (PCC)/precuneus, right temporoparietal junction (RTPJ), and left temporoparietal junction (LTPJ) (Figure 8 and Supplementary Table S4). However, only PCC and RTPJ clusters are present in conjunction analysis (Nichols *et al.*, 2005). We note that searchlight analyses are not independent of the MVPA analysis of the whole DMN ROI and are included as further visualizations of the primary effects reported above.

## Discussion

The present study examined putative overlap in multivariate patterns of activity associated with own and others’ attitudes in the DMN in order to (i) determine whether shared processes are involved in thinking about own and others’ mental states and (ii) improve understanding of egocentrism and consensus bias in social perception. With these ends in mind, classification analyses were conducted for Own and Other Attitude items in order to determine (i) whether information about attitude support or opposition would be present in patterns of DMN activity and (ii) whether such information would be represented similarly in judgments of own and others’ attitudes. The results show that both own and others’ attitudinal positions are encoded within the DMN network, insofar as classification analyses were significant for judgments of both types of attitudes. Moreover, the success of cross-classification analyses (both Own-to-Other and Other-to-Own) implies a significant degree of overlap in the encoding of own and others’ attitudinal positions. Thus, DMN not only encodes own and others’ attitudinal positions, but it appears to do so in similar ways. Lastly, cross-classification accuracy was associated with the magnitude of Own/Other Attitude overlap for each issue. This further result suggests that overlap in neural responses may drive perceived overlap between self and others in attitudes, with greater own/other neural overlap manifesting in the presumption that others share our attitudes.

These findings make an important contribution to our understanding of the shared processes involved in thinking about own and others’ mental states and further extend this work into the attitudinal domain. Not only do we observe activation in common regions within the DMN when thinking about own and others’ attitudes, but multivariate patterns of activation contain similar informational content for self and other, enabling successful classification and cross-classification. It is not conceptually necessary that the neural architecture supporting attitude perception and understanding should be structured in this way; own and others’ attitudinal positions might have been encoded in completely separate brain regions or through different patterns that do not share informational content that would enable cross-classification. The present study thus provides an important conceptual advance in exhibiting the shared nature of these attitudinal representations in the DMN.

### Default egocentrism as a possible consequence of shared DMN representations

Common encoding of own and others’ attitudes in the DMN may also help explain the pervasive tendency towards egocentrism exhibited in consensus estimation (Ross *et al.*, 1977). Our previous work has examined how motivated processes might contribute to the false consensus effect (Welborn *et al.*, 2017; Welborn and Lieberman, 2018). The present study provides an alternative route towards consensus bias in terms of more fundamental overlap in DMN representations of social attitudes: false consensus may occur due to over-reliance on common coding, through a process akin to ‘attribute substitution’ (Kahneman and Frederick, 2002).

When faced with a difficult or complex question, for which crucial information may be unavailable or incomplete, social thinkers often substitute a related but easier question—leading to well-known biases (Tversky and Kahneman, 1974). Social thinkers might substitute their own, readily accessible attitudes and opinions for the unknown and sometimes conflictual attitudes of complex social groups. We theorize that neural usage of common representations and processes for own and others’ attitudes may facilitate this interpersonal attribute substitution, resulting in a form of naïve realism in ordinary social perception (Ross and Ward, 1996). This naïve realism construes attitudes not as mere summary representations of own (or others’) evaluative positions but rather as direct perceptions of objective facts about the world. Our treatment of attitudes may thus be much more akin to ‘seeing’ than to thinking: just as we presume that colors and shapes are ‘out there’ in the world as inalienable features of the objects we perceive, so too do we perceive the evaluative qualities of important social and moral issues to be objective features of our shared social reality. The present results are thus consistent with a model of psychological ‘seeing’ as a pre-reflective, effortless experience, which inhibits perceptual alternatives and appears to the perceiver as given or self-evident (CEEing; see Lieberman, 2022).

Shared representations for self and others may thus help constitute our baseline sense of what is real and valid in the socio-political and moral sphere. If others disagree with us, it is not because their subjective process of attitude formation occurred differently from our own but rather because of their faulty ‘perception’ of socio-political reality. Rather than effortfully examining the differences of opinion, it may be easier to presume that others are biased (Pronin *et al.*, 2004) or susceptible to errors in reasoning (Mata *et al.*, 2013). The resulting ‘bias blind spot’ (Pronin *et al.*, 2002) may reflect this difference in modelling of others’ attitudes based upon our own position. If attitudes are represented by default as (naïve) realist social knowledge about issues and appropriate orientations towards those issues, rather than ascriptions of positions to persons, egocentric projection is a natural consequence (and may be difficult to correct). Our own attitudes, understood as naïve realist perceptions of social reality, may constitute a potent anchor, from which we adjust only effortfully. In this light, the present results are also consonant with prior work, suggesting that MPFC activity reflects anchoring and adjustment in thinking about own and others’ mental states (Tamir and Mitchell, 2010).

### Limitations

The present results do not uniquely identify the causal direction underlying neural and attitudinal overlap as projective, i.e. ‘from’ self ‘to’ others. In many circumstances, simulation of others’ mental states might draw upon the same (or similar) processes involved in reflecting upon our own mental states (Waytz and Mitchell, 2011), especially for similar others (Woo and Mitchell, 2020). However, in other contexts, representations of the self may be influenced by perceptions of others, especially superordinate groups to which we belong (Turner *et al.*, 1987). Attitudinal knowledge may thus be distributed across both general group representations (which include the self) as well as self-specific representations (Karniol, 2003). When the self is not distinctive, we may fall back upon group-based knowledge that applies to both self and others, yielding overlap. Moreover, it is not necessary that overlapping representations in DMN (or other regions) mechanistically precede or cause attitudinal overlap; it is possible that overlapping neural representations coevolve with the alignment of attitudinal positions. Further research will be needed to determine whether changes to neural patterns associated with attitudinal overlap are antecedent or consequent to changes in attitudes. At present, we remain agnostic regarding the specific conditions during which own attitudes may inform perceptions of others’ attitudes and vice versa, and the causal mechanisms associated with the associated changes to self and other representations in the patterns of DMN activity.

We emphasize again that, while consistent with default egocentrism, the results of the present study, in themselves, do not rule out the alternative possibility that individuals’ own attitudes are (chronically) informed by or based upon their perceptions of the attitudes of others (i.e. a default ‘allocentrism’). The results only demonstrate the common encoding of information involved in thinking about own and others’ attitudes. We take the existence of pervasive egocentrism in attitude perception as empirically well-substantiated and view the results as providing a plausible mechanism for this egocentrism, without meeting the higher bar of disconfirming alternatives. In the current study, we feel that egocentric projection is more likely than allocentric assimilation, for two principal reasons. First, we did not explicitly invoke group identity or manipulate situational factors that would be expected to prompt assimilation of own attitudes to others’ attitudes. Second, participants’ estimates of others’ attitudes (relative to the true sample mean) exhibited the familiar error in favor of their own position (i.e. false consensus effect).

The fact that between-subjects classification analyses could generalize attitudinal information across participants has two important implications. First, DMN encoding of positive–negative attitude valence is at least somewhat consistent across persons. Second, the features enabling classification probably do not reflect nuanced, idiosyncratic aspects of attitudinal positions but rather their common evaluative core. This loss of person-specific attitudinal representations may account, in part, for the fact that Own Attitude classifications were not appreciably more accurate than Other Attitude classifications. Person-specific attitudinal representations should be a crucial target for future investigations, and such studies should employ a larger number of attitude items, within subjects, to facilitate the decoding of idiosyncratic attitude content. Moreover, our sample of young, mostly female undergraduates is perhaps more consistent in socio-political attitudes than a random sample of American participants. Between-subject classifications may therefore be less successful with more diverse, heterogeneous samples. Future work will be needed to clarify which structural properties of social attitudes are consistent, and which vary, across individuals.

## Conclusion

The present study contributes to the literature on similarities in mentalizing for self and others, revealing that the DMN encodes own and others’ attitudes using common information. It also provides neuroimaging evidence for a plausible account of own/other attitudinal overlap: our perception of attitudes is grounded in a naïve realism that prioritizes own attitudes. Default egocentrism in social cognition may thus be matched by a corresponding common encoding of own and others’ attitudes in the brain’s DMN. These findings provide a novel explanation of consensus bias in the perception of others’ attitudes and provide a starting point for future research, examining the ways these attitude representations change in response to social processes.

## Supplementary Material

nsad028_Supp

## Data Availability

The data and code that support the findings of this study are available from the corresponding author upon reasonable request.

## Footnotes

1 While Pearson’s correlation coefficient is only marginally significant, all three measures of correlation strength are significant with the removal of the largest multivariate outlier: ‘Banning books’.
