# Supplementary material for: Default egocentrism: an MVPA approach to overlap in own and others’ socio-political attitudes
Source: Soc Cogn Affect Neurosci. 2023 May 27;18(1):nsad028. doi: 10.1093/scan/nsad028 (PMC10281243; doi:10.1093/scan/nsad028)
Supplement: nsad028_Supp [file nsad028_supp.zip › scan-22-015-File010.docx]

**SUPPLEMENTARY MATERIALS:**

**SUPPLEMENTARY METHODS:**

*Own Attitude and Other Attitude Estimation Task – Control and Latitude of Estimation Conditions:*

The *Own Attitude and Other Attitude Estimation Task* also included supplementary conditions, described as follows, in addition to the experimental conditions noted in the primary manuscript. In a control condition, participants were asked to make binary color judgments using a numeric scale similar to that used in assessing Own and Others’ attitudes. On each trial, a colored rectangle appeared, displaying a linear gradient between a pair of colors (drawn from pair-wise combinations of Red, Green, Blue, White, and Black). For example, a trial might display a rectangle that transitioned progressively from pure Green on the left of the screen to pure Red on the right, through intermediate (mixed) values. For each trial, a relevant color target word was presented (e.g. ‘Red’) and participants were asked to indicate the point at which they felt the color of the rectangle transitioned to the color indicated by the target word. This color-judgment condition was intended to control for visual and motor processes involved in scale perception and usage, without invoking evaluative attitudes or perceptions regarding others’ mental states. In a fourth condition, participants indicated the scale-point at which (relative to their own position) the attitude of another individual would become ‘unreasonable’. Because of the complexity of this condition, and because of the present focus on Own/Other attitude estimation, data from this condition are not analyzed herein.

*ROI selection:*

*A priori* ROIs were derived from automated meta-analysis through www.neurosynth.org (Yarkoni et al., 2011), using association-test masks identified by the term ‘default mode’ and exceeding a t-threshold of 7.0. *A priori* ROIs included medial prefrontal cortex (MPFC; peak MNI:-2,50,-6; k=294), precuneus/posterior cingulate cortex (PCC; peak MNI:2,-54,26; k=699), right temporo-parietal junction (RTPJ; peak MNI:50,-62,32; k=366), left temporo-parietal junction (LTPJ; peak MNI:-46,-70,32; k=315), dorsomedial prefrontal cortex (DMPFC; peak MNI:-2,52,24; k=39), left middle temporal gyrus (LMTG; peak MNI:-60,-14,-14; k=42), and right superior frontal gyrus (RSFG; peak MNI:28,28,48; k=28). The union of these voxels was considered for classification analyses using the DMN as a whole.

*SVM Cross-validation Strategy:*

A group shuffle-split strategy based upon scikit-learn was employed to compute cross-validation accuracy for ROI SVM classifications. Importantly, to successfully classify cases (trials) in the testing dataset on each iteration, the classification algorithm must generalize relationships between features (voxels) and attitude item evaluative categories. That is, the classifier must learn from the training set and successfully identify evaluative categories for a test set consisting of data that is entirely novel (for that iteration). This approach is more appropriate than a leave-one-subject-out cross-validation strategy for our data. The leave-one-subject-out approach might have led to unreliable classifications for two reasons: 1) the amount of testing data for each participant is relatively small, and 2) most participants did not have an equal distribution of endorsements across categories (i.e. Opposed, Neutral, Support). The group shuffle-split strategy employed attenuates both these issues, and crucially, no participant is included in both training and testing datasets for any given iteration. Separate randomized training and testing sets were generated across iterations for each classification analysis, in order to minimize the possibility that groupings of participants could help or hinder classification. For all classifications, the default scikit-learn one-vs-rest approach was used for the decision function.

**SUPPLEMENTARY RESULTS:**

*Evaluation of Reaction Time Differences by Condition:*

In order to confirm that basic response properties did not differ drastically across *evaluative* categories (Oppose, Neutral, Support) or *social target* trial type (Own Attitudes, Other Attitudes), reaction times were assessed using a mixed-effects model with participants as a random factor. The mean reaction times for each condition are displayed, with 95% confidence intervals, in *Figure 2D* of the main manuscript. Mean reaction times fell between 5.70s and 6.30s for all conditions. Overall, there was a significant effect of evaluative category on reaction time (F(2,110)=6.413, p=0.00232), but no effect of social target trial type (F(1,110)=0.678, p=0.412) and no interaction (F(2,100)=1.519, p=0.223). Participants were slightly faster to indicate Support than other evaluative categories, for both judgments of Own and Others’ attitudes. Own Support judgments were significantly faster than Own Neutral (t(22)=-3.391, p=0.00262) but not Own Oppose judgments (t(22)=-1.580, p=0.131). Other Support judgments were significantly faster than Other Oppose judgments (t(22)= -3.625, p=0.00150), but not Other Neutral judgments (t(22)= -1.569, p=0.131). Taken together, these results suggest that participants processed Own and Other attitudes similarly, and were slightly faster in making supportive judgments than for other categories.

*Univariate analysis of Default Mode Network activity to Own and Others’ Attitudes:*

As noted in the primary manuscript, mixed-effects models of DMN ROI activity were conducted in order to assess whether hemodynamic activity in the DMN differed by *evaluative* category (Oppose, Neutral, Support) or *social target* trial type (Own Attitudes, Other Attitudes). As expected given the consistent involvement of the DMN in social cognitive paradigms, response to Own and Other attitudes was significantly greater than to control judgments (F(1,137)=121.01, t(137)=11.00, p<0.0001). However, there was no effect of evaluative category (F(2,110)= 0.4255, p= 0.6545) or social target (F(1,100)=2.0608, p=0.1540), or their interaction (F(2,110)= 0.5023, p= 0.6065). For mean BOLD response across conditions (with 95%) confidence intervals, see *Supplementary Figure 1*.

As noted above, there were significant differences in reaction time (RT) for attitude judgments between some of the evaluative categories (e.g. Own Support judgments were faster than Own Neutral judgments, on average). Given these results, we sought to determine whether univariate BOLD activation across social targets (Own/Other) and evaluative categories (Support, Neutral, Oppose) would be affected by RT. To do so, we refit univariate mixed-effects models including RT as a factor (in addition to social target and evaluative category) as well as first-order interactions (with participant as a random factor).

In this full interaction model, there were no main effects of RT (F(1,124.41)= 0.013, p= 0.909), evaluative category (F(2, 105.19)=0.212, p=0.809), or target type (F(1,104.70)=0.1085, p= 0.742). There were no two-way interactions between RT and evaluative category (F(2,105.46=0.144, p=0.866), RT and target type (F(1,104.77)=0.006, p=0.938), or evaluative category and target type (F(2,104.89)=1.172, p=0.314). There was no three-way interaction between all three factors (F(2,104.82)=1.496, p=0.2287). Moreover, a model assessing only RT as a fixed factor (with participant as a random factor) found no effect of RT (F(1,132.5)=0.092, p= 0.762). It is clear that while RT varies slightly between evaluative categories, RT is not a potent predictor of DMN hemodynamic response.

*Univariate analysis of whole-brain activity to Own and Others’ Attitudes:*

While the DMN is the primary focus of the present study (for reasons outlined in the *Introduction*), we also assessed whole-brain univariate response to Own and Other attitude judgment. In particular, we sought to determine 1) whether Own and Other attitudes would evoke activation in similar brain regions, 2) which regions might differentiate between Own and Other Attitudes, and 3) which regions might differentiate between judgments based upon evaluative categories.

The results of this whole-brain analysis are presented in *Supplementary Tables 2* and *3* and *Supplementary Figures 2* and *3*. Relative to the Color control condition, Own and Other attitude judgment elicited widespread activity in numerous regions associated with social cognitive processing, including clusters in the dorsomedial prefrontal cortex (DMPFC), ventromedial prefrontal cortex (VMPFC), the right and left temporoparietal junction (TPJ), the precuneus and posterior cingulate cortex (PCC), the left middle temporal gyrus, and the left temporal pole. Notably, these regions overlap with DMN as defined by automated meta-analysis through Neurosynth ([www.neurosynth.org](http://www.neurosynth.org/); see *Methods* above and *Figure 2C*). Additional regions of activation included the left inferior frontal gyrus as well as posterior visual cortex. The reverse contrasts (Control > Own Attitude and Control > Other Attitude) revealed that some regions were more reliably active in the Control condition than for Own and Other attitude judgments, including the superior parietal lobule, the precentral and postcentral gyri, middle cingulate cortex, and the middle insula. These results are consistent with the extensive literature on the neural correlates of mental state reasoning about self and others (see Lieberman et al., 2019 for a recent review).

Direct contrasts between Own and Other attitude judgment did not reveal differences in brain regions associated with mentalizing. However, Own attitude judgments did elicit greater activation than Other attitude judgments in motor (SMA, left precentral and postcentral gyri) and visual regions (left fusiform gyrus, left middle occipital gyrus). No regions were more responsive, on average, to Other attitude judgments than to Own attitude judgments.

Similarly, whole-brain analysis of univariate activation did not detect consistent differences between evaluative categories in regions associated with mental state reasoning. Supported attitude items evoked greater activity than Neutral items in the right posterior middle temporal gyrus, for both Own and Other attitudes. Opposed items relative to Supported items elicited greater activation in the right temporoparietal junction for Others (but not for Own). See *Supplementary Table 3* and *Supplementary Figure 2* for complete comparisons. It is possible that more complex, quadratic or other curvilinear relationships may hold between attitudinal support and activity in the DMN. Future research should investigate whether non-linear relationships may more effectively capture univariate relationships between DMN activity and attitudinal support (Lebreton, Abitbol, Daunizeau, & Pessiglione, 2015).

Overall, these univariate whole-brain results show much greater consistency than differentiation in attitude-related activity, both for the DMN and other brain regions. Thinking about both Own and Others’ attitudes robustly activated brain regions associated with mental state reasoning and semantic processing. These results are consistent with the ROI analysis above indicating that DMN regions responded similarly to Own and Other attitudes. These regions did not, however, seem to exhibit mean activation level differences based upon evaluative category (Support, Neutral, Oppose).

*Correlations between Attitude Endorsement Proportions and (Cross-)Classification Accuracies:*

For several analyses, Pearon’s, Spearman’s, and Kendall’s Tau correlations are computed between classification (and cross-classification) accuracy and the proportion of attitude items within a given evaluative category (Oppose/Neutral/Support). Several features of these analyses should be noted. First, mean classification accuracies are here computed by attitude item (or issue), averaging over participants. This mean accuracy by attitude item is distinct from the mean accuracy by participant employed elsewhere, but is more useful for visualization. See also *Supplementary Figure 4*, which plots mean classification accuracy for each attitude item for all analyses. Second, proportions of attitude item responses in each evaluative category are also computed separately for each attitude item, reflecting the fraction of participants whose responses fell into that category out of the total number of participants who responded for that attitude item (i.e. excluding non-responses). This approach is necessary, as each participant evaluated each attitude item only once. These proportions are not continuous (i.e. they take on discrete values x/N where x is the number of responses in a given evaluative category and N is the total sample size of responses for that attitude item). As such, the data are best treated as ordinal and analyzed with Spearman’s and Kendall’s Tau correlations. We present Pearson correlation coefficients also for completeness. For the association between own-other overlap and cross-classification accuracy (see *Figure 7*), own-other overlap is computed as the fraction of participants, for each attitude item, whose Own and Other evaluations fell into the same category (e.g. Own Support and Other Support, or Own Oppose and Other Oppose).

We stress that *Figures 4*, *6*, and *7* of the primary manuscript are intended principally as illustrations of the underlying classification and cross-classification analyses. They are not statistically independent of those analyses: that is, given e.g. that classification of Own attitudes by evaluative category is statistically significant, there ought to be an association between the proportion of endorsements in a given evaluative category and the proportion thus classified. These figures thus help to visualize how the DMN supports attitudinal processing, but do not provide independent evidence apart from the classifications that underlie them.

**SUPPLEMENTARY MATERIALS REFERENCES:**

Lebreton, M., Abitbol, R., Daunizeau, J., & Pessiglione, M. Automatic integration of confidence in the brain valuation signal. *Nature Neuroscience* **18**, 1159–1167 (2015). <https://doi.org/10.1038/nn.4064>

Lieberman, M. D., Straccia, M. A., Meyer, M. L., Du, M. & Tan, K. M. Social, self, (situational), and affective processes in medial prefrontal cortex (MPFC): Causal, multivariate, and reverse inference evidence. *Neuroscience & Biobehavioral Reviews* **99**, 311-328 (2019).

Yarkoni, T., Poldrack, T., Nichols, T.E., Van Essen, D.C. & Wager, T.D. Large-scale automated synthesis of human functional neuroimaging data. *Nature Methods* **8**, 665-670 (2011).

**Supplementary Table 1. Attitude Items with Mean Scores and Reaction Times for Own and Other Judgments.** Attitude items were selected for the present study from a larger set of candidate issues, based upon prior behavioral and neuroimaging work on consensus estimation (see main text, *Methods*). The table below indicates, for each attitude item, the mean score for Own Attitude and Other Attitude judgments, the mean reaction time (RT), and the proportions labelled Oppose, Neutral, and Support out of all responses in the present sample.

| Item Name | Mean Value | Mean RT | | Proportion Oppose | Proportion Neutral | Proportion Support |
| --- | --- | --- | --- | --- | --- | --- |
| Own Attitudes: |  |  |  | |  |  |
| Banning books | 19.267 | 5.948 | 0.867 | | 0.067 | 0.067 |
| Polygamous marriages | 29.077 | 6.217 | 0.538 | | 0.385 | 0.077 |
| No sex before marriage | 35.118 | 7.094 | 0.471 | | 0.412 | 0.118 |
| Hunting | 35.813 | 6.326 | 0.625 | | 0.250 | 0.125 |
| Traditional gender roles | 36.667 | 5.885 | 0.533 | | 0.267 | 0.200 |
| Legalized prostitution | 37.800 | 6.217 | 0.467 | | 0.333 | 0.200 |
| Violence in video games | 38.000 | 5.721 | 0.353 | | 0.588 | 0.059 |
| Fossil fuels | 42.625 | 6.280 | 0.438 | | 0.438 | 0.125 |
| Abstinence-based sex education | 44.500 | 6.232 | 0.375 | | 0.438 | 0.188 |
| Right to bear arms | 46.000 | 5.991 | 0.316 | | 0.421 | 0.263 |
| Death penalty | 47.882 | 5.997 | 0.412 | | 0.235 | 0.353 |
| Cloning animals | 48.250 | 5.839 | 0.350 | | 0.300 | 0.350 |
| War on drugs | 50.167 | 5.606 | 0.333 | | 0.389 | 0.278 |
| Stronger border security | 52.769 | 6.142 | 0.308 | | 0.308 | 0.385 |
| Animal research testing | 54.381 | 4.984 | 0.286 | | 0.238 | 0.476 |
| Assisted suicide | 54.867 | 6.313 | 0.267 | | 0.400 | 0.333 |
| Corporate tax cuts / trickle down | 55.400 | 6.734 | 0.333 | | 0.200 | 0.467 |
| Affirmative action | 65.688 | 5.729 | 0.000 | | 0.500 | 0.500 |
| Space flight funding | 70.471 | 5.824 | 0.000 | | 0.529 | 0.471 |
| Foreign aid | 70.471 | 6.035 | 0.000 | | 0.294 | 0.706 |
| Private schools | 71.875 | 6.043 | 0.063 | | 0.250 | 0.688 |
| Embryonic stem cell research | 74.700 | 6.438 | 0.000 | | 0.300 | 0.700 |
| Social welfare programs | 75.947 | 5.295 | 0.000 | | 0.316 | 0.684 |
| Abortion rights | 81.350 | 5.834 | 0.000 | | 0.200 | 0.800 |
| Universal health care | 82.571 | 4.880 | 0.000 | | 0.095 | 0.905 |
| Electric cars | 84.300 | 5.598 | 0.000 | | 0.100 | 0.900 |
| Gay marriage | 85.722 | 5.541 | 0.111 | | 0.000 | 0.889 |
| Organ donation | 86.227 | 5.736 | 0.000 | | 0.091 | 0.909 |
| Recycling | 92.870 | 4.791 | 0.000 | | 0.000 | 1.000 |
| Equal pay for women and men | 93.053 | 5.360 | 0.000 | | 0.000 | 1.000 |
|  |  |  |  | |  |  |
| Other Attitudes: |  |  |  | |  |  |
| Banning books | 15.400 | 6.087 | 0.900 | | 0.050 | 0.050 |
| Polygamous marriages | 23.857 | 6.476 | 0.667 | | 0.333 | 0.000 |
| No sex before marriage | 25.824 | 6.476 | 0.765 | | 0.235 | 0.000 |
| Hunting | 42.529 | 5.747 | 0.353 | | 0.529 | 0.118 |
| Traditional gender roles | 39.050 | 6.003 | 0.450 | | 0.450 | 0.100 |
| Legalized prostitution | 30.824 | 5.724 | 0.647 | | 0.235 | 0.118 |
| Violence in video games | 38.188 | 5.980 | 0.500 | | 0.438 | 0.063 |
| Fossil fuels | 49.444 | 6.118 | 0.333 | | 0.389 | 0.278 |
| Abstinence-based sex education | 39.235 | 6.545 | 0.588 | | 0.294 | 0.118 |
| Right to bear arms | 48.438 | 6.568 | 0.250 | | 0.563 | 0.188 |
| Death penalty | 46.125 | 5.786 | 0.313 | | 0.563 | 0.125 |
| Cloning animals | 48.250 | 5.572 | 0.313 | | 0.438 | 0.250 |
| War on drugs | 47.944 | 5.301 | 0.333 | | 0.500 | 0.167 |
| Stronger border security | 59.647 | 5.762 | 0.000 | | 0.529 | 0.471 |
| Animal research testing | 49.667 | 4.866 | 0.278 | | 0.500 | 0.222 |
| Assisted suicide | 37.950 | 6.349 | 0.550 | | 0.250 | 0.200 |
| Corporate tax cuts / trickle down | 59.000 | 6.203 | 0.214 | | 0.429 | 0.357 |
| Affirmative action | 62.833 | 5.945 | 0.000 | | 0.500 | 0.500 |
| Space flight funding | 69.000 | 6.146 | 0.056 | | 0.333 | 0.611 |
| Foreign aid | 67.118 | 6.275 | 0.000 | | 0.471 | 0.529 |
| Private schools | 65.688 | 6.157 | 0.063 | | 0.375 | 0.563 |
| Embryonic stem cell research | 71.550 | 6.314 | 0.000 | | 0.300 | 0.700 |
| Social welfare programs | 70.400 | 5.424 | 0.050 | | 0.150 | 0.800 |
| Abortion rights | 69.944 | 6.034 | 0.056 | | 0.278 | 0.667 |
| Universal health care | 68.667 | 5.060 | 0.048 | | 0.238 | 0.714 |
| Electric cars | 74.500 | 5.597 | 0.000 | | 0.100 | 0.900 |
| Gay marriage | 71.000 | 5.615 | 0.045 | | 0.227 | 0.727 |
| Organ donation | 75.227 | 5.667 | 0.000 | | 0.182 | 0.818 |
| Recycling | 87.905 | 5.043 | 0.000 | | 0.000 | 1.000 |
| Equal pay for women and men | 83.800 | 5.132 | 0.000 | | 0.100 | 0.900 |
|  |  |  |  | |  |  |

**Supplementary Table 2. Whole-brain univariate analysis of Own/Other activation versus Control**

The table below displays univariate whole-brain analyses of hemodynamic activity during Own/Other attitude estimation, indicating regions in which activity differed for Own or Other attitude estimation relative to control. Tabulated results are corrected for multiple comparisons, cluster FWE p<0.05, with a cluster-formation threshold of p<0.001. Peaks reported are separated by at least 20mm. Because clusters of activation are large, up to 10 peaks are reported per cluster in order to properly distinguish relevant subregions. Conjunction analyses report minimum t-statistic versus conjunction null.

| Test Effect/Anatomical Region | t | x | y | z | k |
| --- | --- | --- | --- | --- | --- |
| Own Attitude > Control: |  |  |  |  |  |
| Left inferior frontal gyrus, orbitalis | 8.092 | -46 | 26 | 2 | 6427 |
| Left temporal pole | 7.660 | -38 | 16 | -24 |  |
| Left middle temporal gyrus | 7.459 | -64 | -14 | -16 |  |
| Dorsomedial prefrontal cortex | 7.098 | -6 | 58 | 22 |  |
|  | 6.782 | -8 | 14 | 70 |  |
|  | 6.647 | -12 | 48 | 44 |  |
|  | 6.591 | -6 | 30 | 56 |  |
| Left inferior frontal gyrus, triangularis | 6.272 | -50 | 22 | 30 |  |
| Left middle frontal gyrus | 6.195 | -34 | 14 | 56 |  |
| Ventromedial prefrontal cortex | 5.969 | -4 | 54 | -10 |  |
| Posterior cingulate cortex | 6.971 | -4 | -52 | 26 | 1912 |
| Lingual gyrus | 6.411 | -18 | -72 | -2 |  |
|  | 5.204 | 6 | -58 | 8 |  |
| Left precuneus | 5.073 | 0 | -66 | 40 |  |
| Calcarine gyrus | 4.784 | -8 | -76 | 20 |  |
|  | 4.290 | 6 | -78 | 2 |  |
| Left middle temporal gyrus | 6.605 | -64 | -44 | 4 | 1482 |
| Left temporoparietal junction | 6.536 | -48 | -60 | 34 |  |
| Right cerebellum | 5.512 | 24 | -76 | -28 | 192 |
| Caudate nucleus | 5.486 | 6 | 10 | 14 | 282 |
|  | 4.556 | -14 | 8 | 16 |  |
| Left lingual gyrus | 4.618 | -18 | -90 | -2 | 114 |
| Left inferior occipital gyrus | 4.264 | -38 | -86 | 0 |  |
| Right temporoparietal junction | 4.532 | 56 | -56 | 38 | 115 |
|  |  |  |  |  |  |
| Own Attitude < Control: |  |  |  |  |  |
| Left superior parietal lobule | -8.884 | -22 | -52 | 68 | 4409 |
| Left postcentral gyrus | -7.612 | -40 | -38 | 60 |  |
| Left precentral gyrus | -6.816 | -28 | -12 | 62 |  |
| Left inferior parietal lobule | -6.599 | -46 | -28 | 42 |  |
| Middle cingulate cortex | -6.180 | -12 | -18 | 46 |  |
| Left postcentral gyrus | -6.025 | -62 | -16 | 40 |  |
| Left superior parietal lobule | -5.138 | -18 | -66 | 48 |  |
| Left Heschl’s gyrus | -4.898 | -46 | -20 | 20 |  |
| Left superior frontal gyrus | -4.257 | -22 | 6 | 48 |  |
| Right postcentral gyrus | -7.963 | 56 | -18 | 38 | 5069 |
| Right superior parietal lobule | -6.972 | 16 | -54 | 60 |  |
| Right postcentral gyrus | -6.470 | 38 | -34 | 50 |  |
| Right middle occipital gyrus | -6.340 | 36 | -64 | 32 |  |
| Right superior occipital gyrus | -6.192 | 18 | -76 | 52 |  |
| Right inferior temporal gyrus | -6.013 | 58 | -54 | -6 |  |
| Right middle temporal gyrus | -5.425 | 36 | -74 | 14 |  |
| Right supramarginal gyrus | -4.493 | 60 | -36 | 46 |  |
| Left middle insula | -7.692 | -38 | -4 | 12 | 457 |
| Left fusiform gyrus | -7.609 | -30 | -50 | -14 | 409 |
| Right superior frontal gyrus | -7.382 | 26 | -2 | 54 | 864 |
| Right fusiform gyrus | -7.339 | 30 | -46 | -14 | 311 |
|  | -3.622 | 10 | -58 | -10 |  |
| Right middle insula | -6.585 | 40 | 2 | 8 | 525 |
|  | -4.833 | 32 | -16 | 4 |  |
|  | -3.985 | 40 | -4 | -12 |  |
| Right inferior frontal gyrus, opercularis | -5.208 | 46 | 0 | 32 | 334 |
| Left precentral gyrus | -5.194 | -52 | 0 | 36 | 138 |
| Middle cingulate cortex | -5.172 | 6 | 10 | 40 | 294 |
| Right middle frontal gyrus | -4.890 | 42 | 52 | 10 | 191 |
|  |  |  |  |  |  |
| Other Attitude > Control: |  |  |  |  |  |
| Dorsomedial prefrontal cortex | 9.038 | 4 | 58 | 26 | 8109 |
| Left middle temporal gyrus | 9.018 | -62 | -38 | 8 |  |
| Left inferior frontal gyrus, triangularis | 8.230 | -52 | 28 | 8 |  |
| Left temporal pole | 8.146 | -44 | 16 | -28 |  |
| Left superior frontal gyrus | 7.942 | -16 | 42 | 44 |  |
| Left middle temporal gyrus | 7.500 | -60 | -14 | -14 |  |
| Superior medial gyrus | 6.821 | -4 | 30 | 60 |  |
| Left inferior frontal gyrus, orbitalis | 6.574 | -42 | 36 | -14 |  |
| Left temporoparietal junction | 6.132 | -48 | -60 | 32 |  |
| Left superior frontal gyrus | 6.042 | -18 | 58 | 26 |  |
| Lingual gyrus | 7.840 | -6 | -68 | 6 | 3308 |
| Posterior cingulate cortex | 7.619 | 8 | -44 | 36 |  |
| Precuneus | 6.992 | -6 | -68 | 32 |  |
|  | 4.457 | -18 | -48 | 8 |  |
| Middle cingulate cortex | 4.413 | 2 | -24 | 38 |  |
| Precuneus | 3.972 | 16 | -68 | 28 |  |
| Right superior frontal gyrus | 6.913 | 22 | 36 | 46 | 353 |
|  | 4.581 | 14 | 26 | 62 |  |
| Left caudate nucleus | 5.695 | -10 | 14 | 10 | 173 |
|  | 4.286 | 6 | 2 | 18 |  |
| Right temporoparietal junction | 5.396 | 48 | -58 | 40 | 348 |
| Right middle temporal gyrus | 4.993 | 50 | -28 | -4 | 453 |
|  | 3.930 | 60 | -12 | -20 |  |
|  |  |  |  |  |  |
| Other Attitude < Control: |  |  |  |  |  |
| Left superior parietal lobule | -12.378 | -22 | -54 | 68 | 8940 |
| Right precentral gyrus | -8.292 | 36 | 0 | 56 |  |
| Left postcentral gyrus | -8.225 | -46 | -30 | 58 |  |
| Right middle insula | -7.895 | 38 | 0 | 12 |  |
| Left precentral gyrus | -7.620 | -36 | -10 | 58 |  |
| Left postcentral gyrus | -7.542 | -64 | -12 | 36 |  |
| Left middle occipital gyrus | -6.597 | -38 | -76 | 16 |  |
| Left rolandic operculum | -6.385 | -42 | -26 | 20 |  |
| Middle cingulate cortex | -6.254 | -2 | 8 | 48 |  |
| Right inferior frontal gyrus, opercularis | -5.820 | 54 | 10 | 32 |  |
| Right cerebellum | -8.749 | 18 | -48 | -18 | 760 |
| Right postcentral gyrus | -8.335 | 58 | -20 | 36 | 5176 |
| Right superior parietal lobule | -6.757 | 28 | -58 | 62 |  |
| Right middle temporal gyrus | -6.691 | 48 | -68 | 16 |  |
| Right middle cingulate cortex | -6.647 | 18 | -42 | 50 |  |
| Right superior occipital gyrus | -6.152 | 26 | -68 | 44 |  |
| Right inferior temporal gyrus | -5.398 | 48 | -62 | -6 |  |
| Right postcentral gyrus | -5.366 | 42 | -34 | 50 |  |
| Right middle occipital gyrus | -4.277 | 30 | -84 | 30 |  |
| Left cerebellum | -7.518 | -32 | -48 | -30 | 844 |
|  | -5.501 | -30 | -68 | -14 |  |
|  | -3.630 | -14 | -54 | -12 |  |
| Left middle insula | -6.702 | -38 | -4 | 12 | 243 |
| Left middle temporal gyrus | -5.880 | -46 | -68 | 4 | 340 |
| Left inferior frontal gyrus, opercularis | -5.733 | -62 | 10 | 18 | 261 |
|  | -4.643 | -54 | 4 | 36 |  |
|  |  |  |  |  |  |
| Conjunction, Own Attitude > Control $\cap$ Other Attitude > Control: |  |  |  |  |  |
|  |  |  |  |  |  |
| Left inferior frontal gyrus, orbitalis | 7.396 | -44 | 28 | 0 | 2373 |
| Left middle temporal gyrus | 6.948 | -62 | -14 | -14 |  |
| Left temporal pole | 6.504 | -46 | 12 | -30 |  |
| Left inferior frontal gyrus, triangularis | 5.799 | -50 | 22 | 28 |  |
| Left middle frontal gyrus | 4.547 | -34 | 18 | 46 |  |
| Left insula | 4.096 | -26 | 14 | -14 |  |
| Posterior cingulate cortex | 6.816 | -4 | -52 | 26 | 1377 |
| Lingual gyrus | 5.932 | -6 | -68 | 6 |  |
| Precuneus | 5.073 | 0 | -66 | 40 |  |
| Superior medial gyrus | 6.697 | -6 | 30 | 60 | 2156 |
| Dorsomedial prefrontal cortex | 6.623 | -4 | 58 | 24 |  |
|  | 6.154 | -16 | 42 | 42 |  |
|  | 4.719 | 8 | 50 | 48 |  |
| Medial prefrontal cortex | 4.375 | 2 | 60 | 4 |  |
| Left middle temporal gyrus | 6.605 | -64 | -44 | 4 | 1346 |
| Left temporoparietal junction | 6.132 | -48 | -60 | 32 |  |
|  | 4.947 | -44 | -62 | 52 |  |
| Right superior frontal gyrus | 5.184 | 20 | 40 | 50 | 201 |
|  |  |  |  |  |  |
| Conjunction, Own Attitude < Control $\cap$ Other Attitude < Control: |  |  |  |  |  |
| Left superior parietal lobule | -8.884 | -22 | -52 | 68 | 3600 |
| Left precentral gyrus | -6.423 | -26 | -4 | 54 |  |
| Left postcentral gyrus | -6.322 | -40 | -36 | 58 |  |
| Left inferior parietal lobule | -6.128 | -52 | -26 | 44 |  |
| Left superior parietal lobule | -5.033 | -18 | -68 | 50 |  |
| Left rolandic operculum | -4.898 | -46 | -20 | 20 |  |
| Left postcentral gyrus | -4.473 | -34 | -40 | 38 |  |
| Right postcentral gyrus | -7.547 | 58 | -18 | 38 | 4010 |
| Right superior parietal lobule | -6.357 | 22 | -54 | 58 |  |
| Right middle occipital gyrus | -5.682 | 40 | -70 | 30 |  |
| Right postcentral gyrus | -5.366 | 42 | -34 | 50 |  |
| Right inferior temporal gyrus | -4.772 | 58 | -60 | -8 |  |
| Right middle temporal gyrus | -4.652 | 48 | -64 | 12 |  |
| Left fusiform gyrus | -7.356 | -28 | -52 | -12 | 370 |
| Right fusiform gyrus | -7.284 | 28 | -48 | -16 | 251 |
| Right cerebellum | -3.622 | 10 | -58 | -10 |  |
| Right precentral gyrus | -7.233 | 24 | -2 | 54 | 731 |
| Left middle insula | -6.702 | -38 | -4 | 12 | 190 |
| Right middle insula | -6.585 | 40 | 2 | 8 | 230 |
| Right precentral gyrus | -5.191 | 46 | 0 | 32 | 250 |
| Middle cingulate cortex | -5.055 | 4 | 8 | 42 | 184 |
|  |  |  |  |  |  |

**Supplementary Table 3. Whole-brain univariate analysis of Own/Other activations across attitude Evaluative Category Levels (Oppose, Neutral, Support)**

The table below displays univariate whole-brain analyses of hemodynamic activity during Own/Other attitude estimation, indicating regions in which activity differed based upon the levels of endorsement (Oppose, Neutral, Support). Tabulated results are corrected for multiple comparisons, cluster FWE p<0.05, with a cluster-formation threshold of p<0.001. Peaks reported are separated by at least 20mm. Up to three peaks are reported per cluster.

| Test Effect/Anatomical Region | t | x | y | z | k |
| --- | --- | --- | --- | --- | --- |
| Own Support > Own Neutral: |  |  |  |  |  |
| Right middle temporal gyrus | 9.5562 | 50 | -66 | 10 | 351 |
|  |  |  |  |  |  |
| Own Support < Own Neutral: |  |  |  |  |  |
| None |  |  |  |  |  |
|  |  |  |  |  |  |
| Other Support > Other Neutral: |  |  |  |  |  |
| Left middle cingulate cortex | 5.8174 | -2 | 4 | 44 | 406 |
| Right posterior-medial frontal | 5.5632 | 10 | -4 | 58 |  |
| Right middle temporal gyrus | 5.6197 | 58 | -66 | 10 | 239 |
| Right postcentral gyrus | 5.4343 | 60 | -18 | 26 | 122 |
|  |  |  |  |  |  |
| Other Support < Other Neutral: |  |  |  |  |  |
| None |  |  |  |  |  |
|  |  |  |  |  |  |
| Own Support > Own Oppose: |  |  |  |  |  |
| Right middle temporal gyrus | 4.6504 | 50 | -66 | 4 | 63 |
|  |  |  |  |  |  |
| Own Support < Own Oppose: |  |  |  |  |  |
| None |  |  |  |  |  |
|  |  |  |  |  |  |
| Other Support > Other Oppose: |  |  |  |  |  |
| None |  |  |  |  |  |
|  |  |  |  |  |  |
| Other Support < Other Oppose: |  |  |  |  |  |
| Right temporo-parietal junction | -4.6025 | 44 | -60 | 24 | 189 |
|  |  |  |  |  |  |
|  |  |  |  |  |  |
| Own Neutral > Own Oppose: |  |  |  |  |  |
| None |  |  |  |  |  |
|  |  |  |  |  |  |
| Own Neutral < Own Oppose: |  |  |  |  |  |
| Right precuneus | -5.1552 | 20 | -76 | 52 | 115 |
|  |  |  |  |  |  |
| Other Neutral > Other Oppose: |  |  |  |  |  |
| None |  |  |  |  |  |
|  |  |  |  |  |  |
| Other Neutral < Other Oppose: |  |  |  |  |  |
| Right temporo-parietal junction | -5.6775 | 46 | -56 | 20 | 341 |
|  | -4.4123 | 58 | -60 | 0 |  |
| Right superior frontal gyrus | -5.3576 | 18 | -8 | 68 | 171 |
| Right postcentral gyrus | -4.7192 | 64 | -22 | 36 | 146 |
|  |  |  |  |  |  |

**Supplementary Table 4. DMN MVPA searchlight using 6mm spherical ROIs, classifying Own and Other Attitudes by Evaluative Category Level (Oppose, Neutral, Support)**

The table below displays results of multivariate searchlight analyses of hemodynamic activity from 6mm spherical searchlight ROIs within the DMN. Peak coordinates and accuracy scores are tabulated for three-way classifications of own and other attitudes by levels of endorsement (Oppose, Neutral, Support). Results are significant at p<0.001 uncorrected, using permutation testing with a minimum cluster extent of 15 voxels. Peaks reported are separated by at least 20mm. Up to three peaks are reported per cluster.

| Test Effect/Anatomical Region | Accuracy: | x | y | z | k |
| --- | --- | --- | --- | --- | --- |
| Own Attitudes [Oppose/Neutral/Support]: |  |  |  |  |  |
| Medial prefrontal cortex | 0.454 | -2 | 50 | 2 | 84 |
| Right temporo-parietal junction | 0.440 | 48 | -66 | 38 | 151 |
| Dorsomedial prefrontal cortex | 0.438 | 0 | 50 | 26 | 28 |
| Posterior cingulate cortex | 0.434 | 0 | -42 | 36 | 154 |
|  | 0.427 | 6 | -50 | 18 |  |
| Medial prefrontal cortex | 0.428 | 0 | 58 | 6 | 30 |
| Left temporo-parietal junction | 0.410 | -48 | -68 | 36 | 24 |
|  |  |  |  |  |  |
| Other Attitudes [Oppose/Neutral/Support]: |  |  |  |  |  |
| Precuneus | 0.438 | -2 | -54 | 34 | 168 |
|  | 0.407 | 2 | -58 | 14 |  |
| Right temporo-parietal junction | 0.425 | 56 | -62 | 36 | 64 |
| Left temporo-parietal junction | 0.421 | -42 | -70 | 32 | 33 |
| Right temporo-parietal junction | 0.403 | 46 | -58 | 36 | 16 |
|  |  |  |  |  |  |
| Conjunction, Own Attitudes $\cap$ Other Attitudes [Oppose/Neutral/Support]: |  |  |  |  |  |
| Right temporo-parietal junction | 0.437 | 56 | -62 | 36 | 36 |
| Precuneus | 0.422 | -6 | -60 | 30 | 16 |
|  |  |  |  |  |  |

**SUPPLEMENTARY FIGURES:**


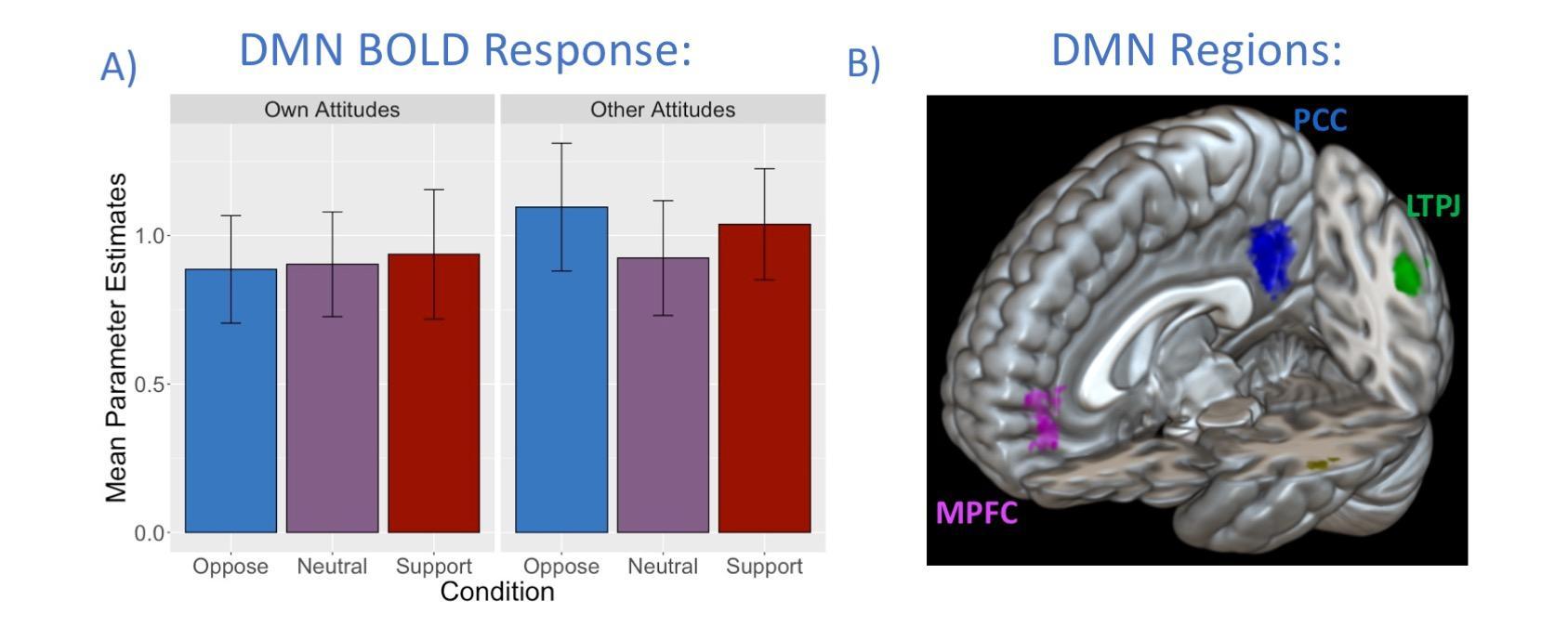


**Supplementary Figure 1:** Univariate region-of-interest (ROI) analysis of Own and Other Attitudes. (A) Mean univariate activation in the DMN is displayed for each condition relative to the color judgment control task, with associated 95% confidence intervals. The mean level of hemodynamic response did not differ between targets or evaluative categories. (B) Regions of the DMN were identified based upon automated association-test meta-analysis with Neurosynth ([www.neurosynth.org](http://www.neurosynth.org/)) exceeding a threshold of t=7.0: medial prefrontal cortex (MPFC; pink), posterior cingulate/precuneus (PCC; blue), left temporoparietal junction (LTPJ; green); right temporoparietal junction (RTPJ; not shown), and left and right middle temporal gyrus (not shown).


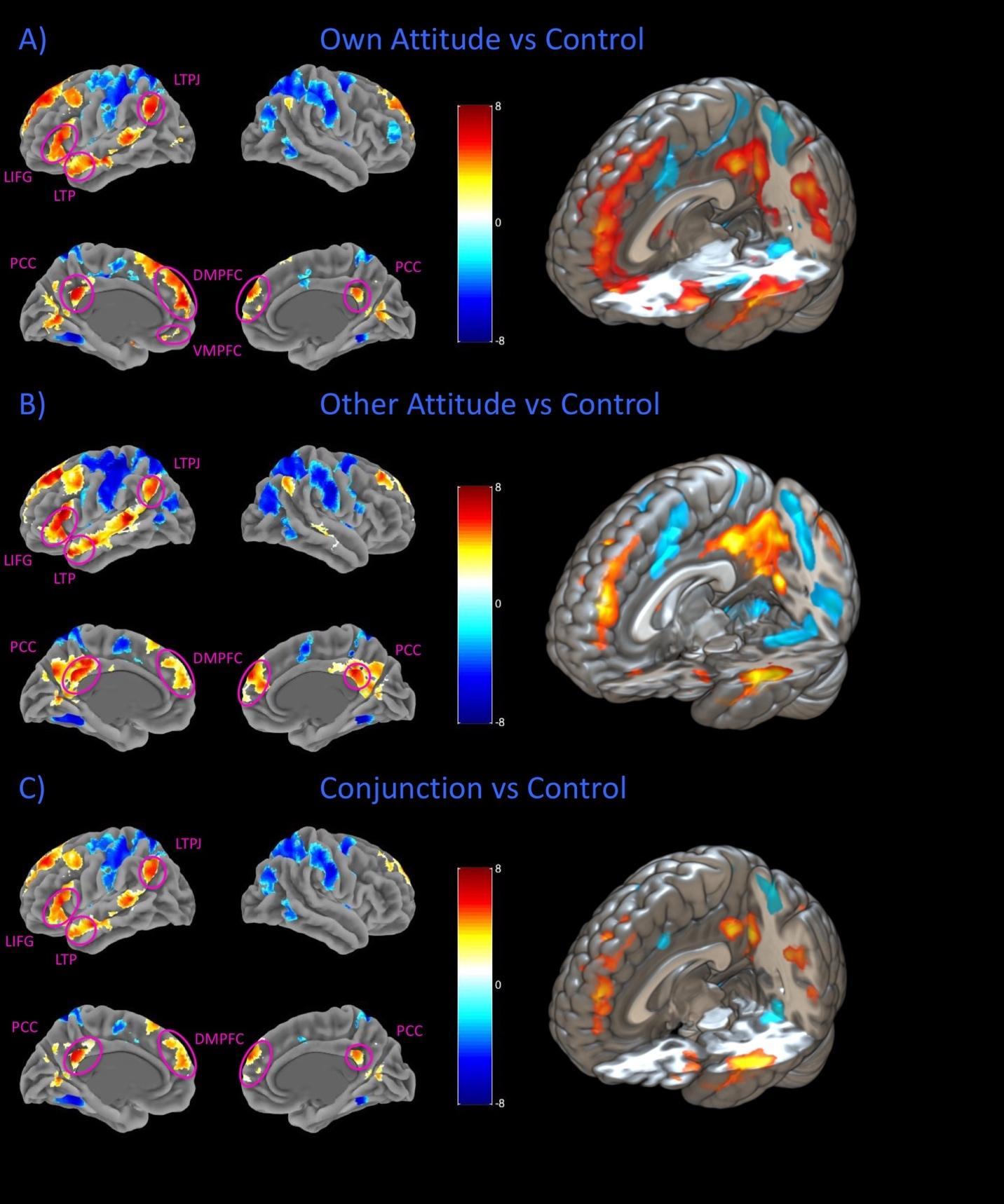


**Supplementary Figure 2:** Univariate GLM models of hemodynamic (BOLD) response reveal consistent activation during both Own Attitude expression (A) and Other Attitude estimation (B), relative to the Control (color judgment) task. Conjunction analysis (minimum statistic versus conjunction null) shows regions active in both Own and Other judgments (C). Both types of attitude judgments elicit activity in canonical DMN regions, including the MPFC, TPJ, and PCC/precuneus. Several regions are also deactivated relative to attitude judgment (see *Supplementary Table 2*). Analyses are corrected for multiple comparisons with FWE p<0.05, using a cluster-formation threshold of p<0.001. DMPFC: Dorsomedial prefrontal cortex; VMPFC: ventromedial prefrontal cortex; PCC: posterior cingulate cortex; LTPJ: left temporo-parietal junction; LTP: left temporal pole; LIFG: left inferior frontal gyrus.


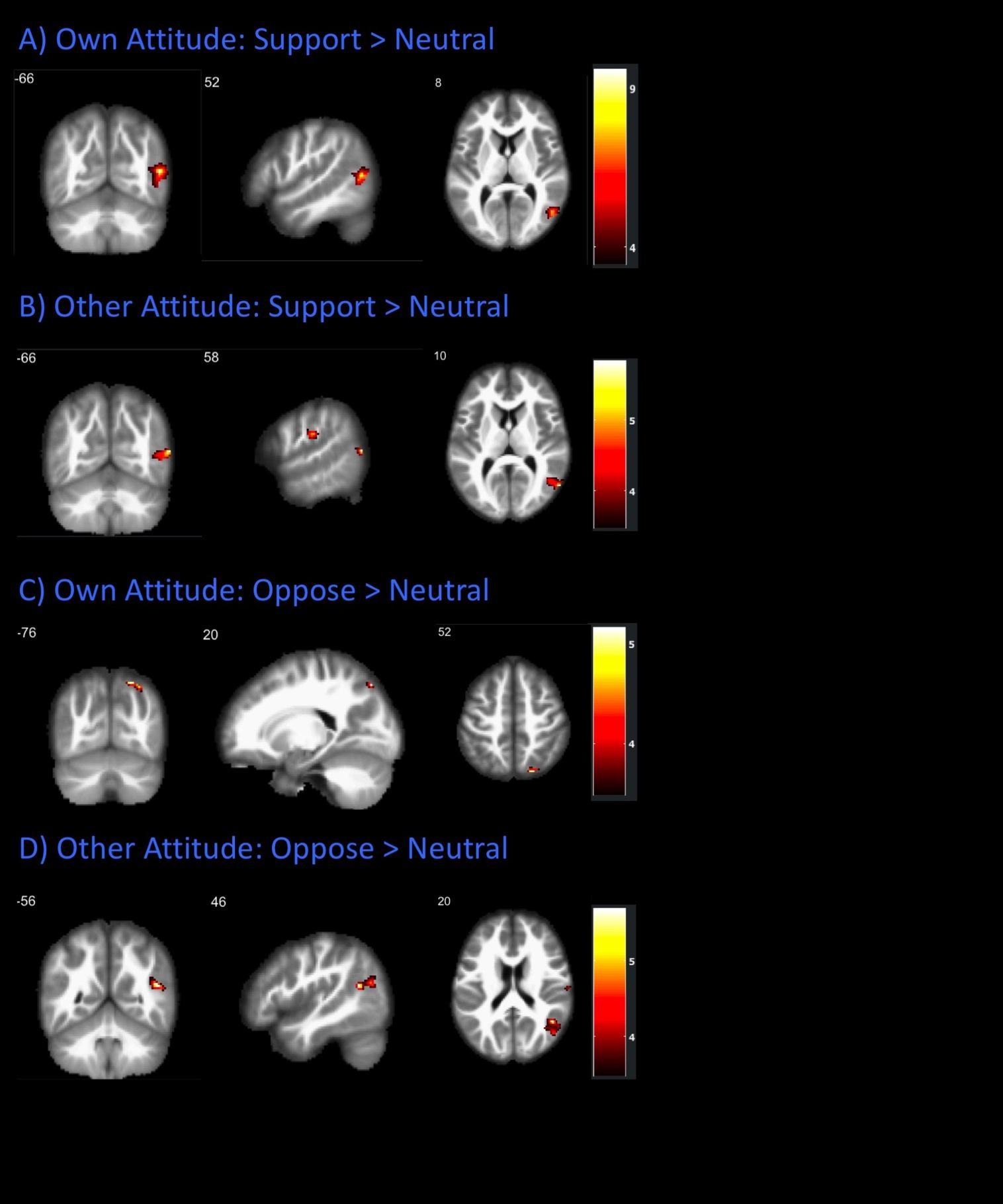


**Supplementary Figure 3:** Univariate GLM analysis shows that several regions differ between levels of endorsement (Oppose, Neutral, Support) for Own Attitude and Other Attitude judgments. Selected contrasts depicted above focus on differences between the relatively extreme items (Oppose and Support) and the Neutral attitude items (see also *Supplementary Table 2*). Supported items evoked elevated activity in the right middle temporal gyrus relative to Neutral items, for both Own (A) and Other judgments (B). The right superior parietal lobule was more engaged for Own judgments when attitudes were judged to be Opposed by the ordinary person relative to Neutral judgments (C). The right temporo-parietal junction was more engaged for Other judgments when attitudes were judged to be Opposed by the ordinary person relative to Neutral judgments (D) and Supported judgments. Analyses are corrected for multiple comparisons with FWE p<0.05, using a cluster-formation threshold of p<0.001. Sections display peak coordinates for the clusters depicted; note that not all clusters from *Supplementary Table 2* appear above.


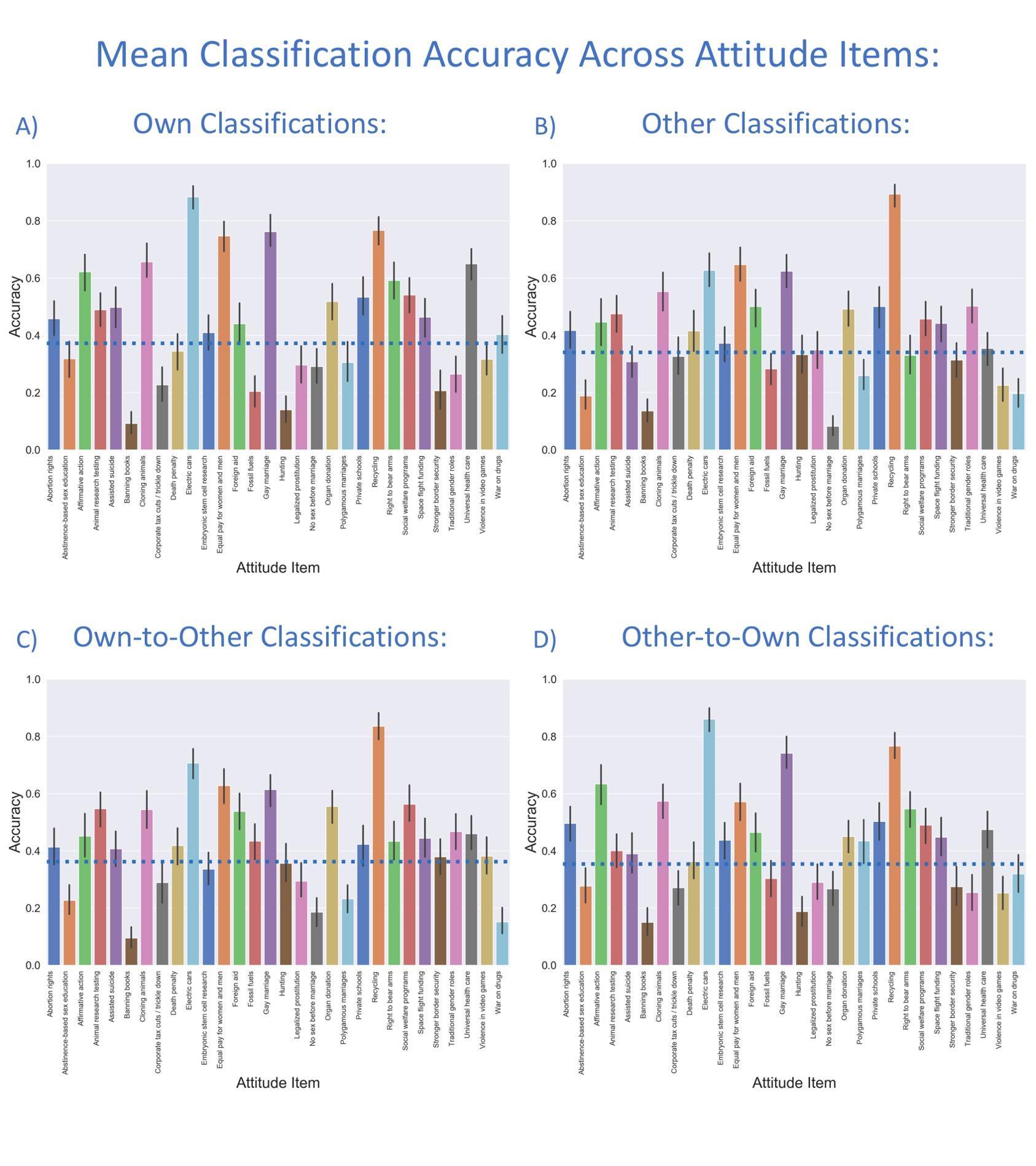


**Supplementary Figure 4:** For each of the four primary DMN classifications, accuracy varied considerably across attitude items (i.e. across socio-political issues). The bar charts above plot the mean classification accuracy for each item (with associated 95% confidence intervals), for (A) Own Attitude classifications, (B) Other Attitude classifications, (C) Own-to-Other cross-classifications, and (D) Other-to-Own reverse cross-classifications. Chance classification accuracies (based on permutation tests with shuffled evaluative category labels) are indicated by the dotted blue lines. Accuracy is at or above chance for the majority of attitude items for each of the four classifications. Interestingly, classification accuracy appears to be lowest for issues stereotypically associated with political conservatism. See also *Figures 3* and *5* of the primary manuscript.
